# No evidence of transposable element bursts in the Galápagos *Scalesia* adaptive radiation despite hybridization, diversification and ecological niche shifts

**DOI:** 10.1186/s13100-025-00362-z

**Published:** 2025-05-31

**Authors:** José Cerca, Patricia Jaramillo Díaz, Clément Goubert, Heidi Yang, Vanessa C. Bieker, Mario Fernández-Mazuecos, Pablo Vargas, Rowan Schley, Siyu Li, Juan Ernesto Guevara-Andino, Bent Petersen, Gitte Petersen, Neelima R. Sinha, Lene R. Nielsen, James H. Leebens-Mack, Gonzalo Rivas-Torres, Loren H. Rieseberg, Michael D. Martin

**Affiliations:** 1 Department of Natural History, University Museum, Norwegian University of Science and Technology (NTNU), Trondheim, Norway; 2 Centre for Ecological and Evolutionary Synthesis (CEES), Department of Biosciences, University of Oslo, Oslo, Norway; 3 Department of Bioinformatics and Genetics, Swedish Museum of Natural History, Stockholm, Sweden; 4 SciLifeLab, Karolinska Institutet Science Park, Tomtebodavägen 23, Solna, 171 65 Sweden; 5 Estación Científica Charles Darwin, Fundación Charles Darwin, Santa Cruz, Galápagos, Ecuador; 6 Department of Botany and Plant Physiology, University of Málaga, Málaga, Spain; 7 IUCN SSC Galapagos Plant Specialist Group, Puerto Ayora, Galapagos, 200102 Ecuador; 8 McGill Genome Centre, McGill University, Montreal, QC H3A 0G1 Canada; 9 Department of Ecology & Evolutionary Biology, University of California, Los Angeles, Los Angeles, CA USA; 10 Real Jardín Botánico (RJB), CSIC, Madrid, ES Spain; 11 Department of Biodiversity and Conservation, Real Jardín Botánico de Madrid (RJB-CSIC), Madrid, 28014 Spain; 12 Department of Geography, University of Exeter, Laver Building, North Park Road, Exeter, Devon, UK; 13 Department of Plant Biology, University of California, Davis, Davis, CA 95616 USA; 14 Grupo de Investigación en Ecología y Evolución en los Trópicos-EETrop, Universidad de las Américas, Quito, Ecuador; 15 Center for Evolutionary Hologenomics, Globe Institute, University of Copenhagen, Copenhagen, DK-1353 Denmark; 16 Centre of Excellence for Omics-Driven Computational Biodiscovery (COMBio), Faculty of Applied Sciences, AIMST University, Kedah, Malaysia; 17 Department of Ecology, Environment and Plant Sciences, Stockholm University, Stockholm, 106 91 Sweden; 18 Department of Geosciences and Natural Resource Management, University of Copenhagen, Rolighedsvej 23, Frederiksberg C, 1958 Denmark; 19 Department of Plant Biology, University of Georgia, Athens, GA 30602 USA; 20 Colegio de Ciencias Biológicas y Ambientales, Galapagos Science Center, Universidad San Francisco de Quito USFQ, 170901 Quito, Ecuador; 21 Department of Botany and Biodiversity Research Centre, University of British Columbia, Vancouver, BC V6T 1Z4 Canada; 22 R. Ken Coit College of Pharmacy, University of Arizona, Tucson, AZ USA; 23 Jardín Botánico (RJB), CSIC, Madrid, Spain

## Abstract

Transposable elements (TEs) have been hypothesized to play a pivotal role in driving diversification by facilitating the emergence of novel phenotypes and the accumulation of divergence between species. Hybridization and adaptation to novel niches have been proposed to destabilize mechanisms constraining TE proliferation, potentially inducing a ‘TE burst’ that promotes TE accumulation on the genome. The rapid speciation and ecological diversification characteristic of adaptive radiations offer a unique opportunity to examine the link between TE accumulation and speciation, diversification, hybridization and adaptation. Here, focusing on all 15 species of the genus *Scalesia* (Asteraceae), a radiation endemic to the Galápagos Islands, we test whether diversification, hybridization, or shifts in ecological niche are associated with changes in TE accumulation in genomes. Our analyses reveal little to no variation in TE accumulation among *Scalesia* species nor its hybrid populations. Shifts in ecological niches, linked to climatic variation, did not result in discernible changes in TE accumulation, a surprising finding given the anticipated selective pressure imposed by aridity, a factor often linked to genome size reduction. We found no distinct patterns in the temporal accumulation of TEs, and no effects at the class or superfamily level. Our findings suggest that while TEs may play a key role in evolution at the locus level, their macroevolutionary association with diversification or speciation appears weak. Rather than actively driving evolutionary diversification, TEs may simply be'along for the ride.

**Supplementary Information:**

The online version contains supplementary material available at 10.1186/s13100-025-00362-z.

## Introduction

Transposable elements (TEs) are mobile DNA sequences that can relocate within a genome [1]. Through their replication and mobility, TEs generate diverse mutations, including alterations in promoter/enhancer gene sequences, frameshifts, intronic modifications and gene duplications within their hosts [2–8]. Some of these mutations have been directly linked to the evolution of novel phenotypes. A notable example is the evolution of the melanic phenotype in the peppered moth (*Biston betularia*)–the classical textbook example of adaptation– where the insertion of the carb-TE into an intronic region of the cortex gene alters its expression, likely contributing to the dark coloration [9].

Advances in genomic sequencing technology and processing algorithms have greatly facilitated the detection and characterization of TEs, particularly in non-model organisms with limited genomic resources [10]. The evident adaptive potential of TEs in shaping phenotypes [4, 11, 12], such as for the peppered moth, along with the evidence of substantial variation in TE diversity and accumulation across different reference genomes (e.g [13, 14])., has intensified interest in a potential relationship between TEs and diversification. ​​One particularly speculative hypothesis is that lineages with high diversification rates also exhibit elevated rates of TE accumulation on their genomes, as a result of TE bursts [6, 15–22]. For instance, Falcon et al. (2023) hypothesize that TE expansions played a significant role in enabling major evolutionary radiations, such as the water-to-land transition, by enriching cis-regulatory elements with repeat sequences. Oliver and Greene (2009) further propose that episodic surges of TE activity can explain punctuated equilibrium-dynamics and facilitate rapid evolutionary change, particularly in species-rich lineages like rodents and bats, which exhibit high TE activity. Pimpinelli et al. (2019) extend this idea by suggesting that environmental changes can induce TE activation, generating genetic variability that underpins rapid adaptive speciation, as seen in Hawaiian *Drosophila*. Similarly, de Boer et al. (2007) suggest that bursts of transposon replication activity coincided with the radiation and speciation of salmonid fishes, highlighting the potential for TEs to drive lineage divergence. Belyayev (2014) emphasizes that TE bursts can provoke radical genomic rebuilding, often associated with the genesis of new phylogenetic groups. Ricci et al. (2018) further support this by showing that mammalian taxa with high speciation rates are associated with “hot” genomes characterized by active TEs, whereas those with low speciation rates have “cold” genomes. A key limitation of these studies has been their reliance on a small number of genomes, often with limited taxonomic coverage while encompassing large clades, and primarily based on correlations. While we recognize that locus-specific effects of TE insertions play a significant role in the evolution of novel phenotypes, the evidence linking global bursts of TEs to diversification may be overstated.

Adaptive radiations represent evolutionary experiments where extensive ecological variation aligns with rapid speciation, and they have been a cornerstone of our understanding of evolutionary processes and mechanisms [23–25]. Adaptive radiations can also yield valuable insights into the interplay between TEs and evolutionary success; however, the evidence available is conflicting and largely originated from cichlid radiations. For example, a study examining four species within an African cichlid adaptive radiation found higher TE counts in the genomes and transcriptomes of the radiating species compared to non-radiating lineages [26]. More recent evidence, however, suggested a lack of correlation between species richness at the tribe level in the Lake Tanganyika adaptive radiation and TE content across 245 genomes [27]. Cichlid lineages in the Americas had no discernible differences in TE content when contrasting a radiating lineage (*Amphilophus citrinellus*) with a non-radiating lineage (*Archocentrus centrarchus*; [28]. This conflicting body of evidence underscores the necessity for further exploration of TEs as facilitators of diversifications, particularly extending beyond the scope of cichlid radiations.

**Fig. 1:**
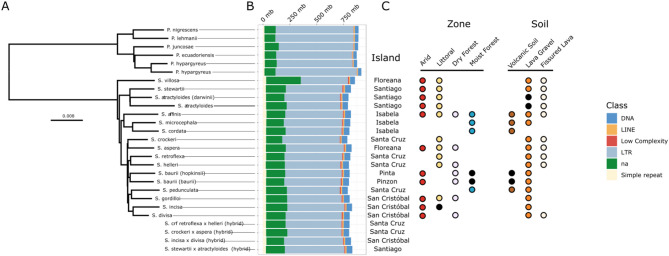
Phylogenetic relationships and transposable element (TE) variation in the *Scalesia* radiation and the outgroup (genus *Pappobolus*). (**a**) Neighbor-joining phylogeny of *Scalesia* and the outgroup. Hybrid populations are shown below and were not included in the phylogenetic reconstruction. (**b**) Stacked bar plot illustrating TE and repeat groups (for the analysis displayed here we used the sunflower library; the analysis using a *Scalesia*-based library is found in Supplementary Fig. 2). Color-coding of the different repeat and TE groups is found in the rightmost part of the figure, where ‘na*’* denotes unidentified TEs. (**c**) Labelling of the different islands, environments, and soils occupied by different *Scalesia* species (for hybrids we include only island labelling). Environment and soil labelling follow [30], and black dots indicate uncertainty in environmental characterization

The *Scalesia* radiation (Asteraceae, Asterales, Magnoliidae), endemic to the Galápagos archipelago, presents an excellent opportunity to investigate the potential relationship between TEs and diversification. This genus comprises ~ 15 species that evolved from a single common ancestor which diversified about 1 million years ago [29]. The recent origin of this radiation facilitates the reconstruction and tracking of TE accumulation over time. Furthermore, *Scalesia* exhibits remarkable ecological diversity, particularly in its occupation of different soil types and various climate zones (*hereafter* referred to as climate niches; Fig. 1) [30]. These niches are characterized by large ecological differences involving differences in saltwater exposure, aridity, precipitation, temperature, intense solar radiation. This ecological variation provides an opportunity to explore whether transitions betwen climatic correlate with TE dynamics [14]. Specifically, because the accumulation of TEs can result in an increase in genome size, which is associated with larger stomatal cells with impaired gas exchange efficiency and poor water economy [31], natural selection may regulate genome size in species adapted to arid conditions. Furthermore, hybridization has been documented in this radiation through genomic analyses and the identification of morphological intermediate populations [29]. This provides an opportunity to test a long-standing but rarely observed hypothesis: whether hybridization triggers genomic instability by activating TEs [32–38]. Finally, the reference genome assembly for the group, ideal to understand TE accumulation dynamics [39].

Building on the premise that TEs play a role in diversification dynamics, here we test three hypotheses: *(H1)* Lineages undergoing rapid speciation and broadening of ecological variation exhibit an increased rate of lineage- or taxon-specific TE accumulation/expansion on their genomes; *(H2)* Shifts in climatic niches result in a change of selective regimes, with lineages in arid areas experiencing stronger selection for TE removal as a result of selection for smaller cells; and *(H3)*. Hybrids have an increased accumulation of TEs as a result of genome deregulation. To test these hypotheses, we employed whole-genome resequencing of all 15 *Scalesia* species, four *Scalesia* morphologically intermediate populations (‘hybrid populations’), and outgroup species, integrating genomic results with ecological data [30, 40].

## Materials and methods

### Study species and data collection

*Scalesia* (Asteraceae) is an endemic genus to the Galápagos Islands consisting of approximately 15 species derived from a single common ancestor. The divergence of *Scalesia* from members of a continental sister lineage, *Pappobolus*, occurred around 3 million years ago, while most diversification of the group occurred within the last million years [29]. *Scalesia* occupies a wide array of ecological niches and soil types (Fig. 1) [30], displaying clear environment-phenotype associations [29, 39], as expected in an adaptive radiation [25]. In this study, we sequenced samples representing the entire *Scalesia* genus, including: *S. affinis*,* S. aspera*,* S. atractyloides* (var. *darwinii*), *S. baurii* (subsp. *baurii* and *hopkinsii*), *S. cordata*,* S. crockeri*,* S. divisa*,* S. gordilloi*,* S. helleri*,* S. incisa*,* S. microcephala*,* S. pedunculata*,* S. retroflexa*,* S. stewartii*, and *S. villosa* (Supplementary Table 1). Additionally, we carried out sequencing of samples from populations that show evidence of intermediate morphologies, likely as a result of hybridization (hereafter “hybrid population”). The hybridizing populations included only pairs of sister species: *S. retroflexa* × *helleri*, *S. crockerii* × *aspera*, *S. incisa* × *divisa*, and *S. stewartii* × *atractyloides* (Supplementary Table 1). We incorporated individuals from the genus *Pappobolus* (sister group to *Scalesia*) from the South American continent as the outgroup in some analyses [29, 30], including *P. ecuadoriensis*, *P. hypargyreus*, *P. juncosae*, *P. lehmanii*, and *P. nigrescens*. Detailed information and accession numbers regarding the sampling of outgroup species can be found in Fernández-Mazuecos et al. (2020). All samples used are from the Charles Darwin Herbarium or the Copenhagen Herbarium.

### Data generation (library preparation and sequencing)

We extracted genomic DNA using the DNeasy 96 Plant Kit (Qiagen) following the manufacturer’s protocol with minor modifications. These modifications consisted of grinding dried leaf tissues in a Qiagen collection microtube with a 3-mm tungsten carbide bead and repeating the membrane washing step with an additional 800 µl of buffer AW2 to remove residual greenish color from the samples. We eluted the DNA from the membranes using 50 µl of buffer AE to obtain a high-concentration DNA yield. The DNA extracts were then sent to the commercial provider Novogene UK for shearing to a mean insert size of 350 bp, double-stranded DNA genomic library preparation, indexing and amplification via PCR, and 150-bp paired-end sequencing on the Illumina NovaSeq 6000 platform. The average sequencing depth was ~ 6x. Three samples used in this study were obtained from the Charles Darwin Research station herbarium and were prepared for genomic sequencing independently. Leaf tissue was disrupted using a Tissue Lyser II (Quiagen), followed by DNA extraction using the DNeasy Plant Mini Kit following the manufacturer’s protocol except for an additional overnight incubation step with 20 µL of proteinase K after cell lysis as described in [41]. DNA was eluted using 62 µL buffer AE. Extracts were then sent to the commercial provider Novogene for double-stranded DNA genomic library preparation, indexing and amplification via PCR, and 150-bp paired-end sequencing on the Illumina NovaSeq 6000 platform. The raw sequencing data generated for this study can be found at the European Nucleotide Archive under accession code PRJEB70770.

### Low-coverage phylogenomics

We started by conducting a distance-based phylogenetic reconstruction to investigate the evolutionary history of the *Scalesia* genus and to establish a backbone for subsequent analyses and interpretations. This analysis was conducted to compare the resulting topology with that obtained by [29] and to assess whether hybrid populations were positioned as sister groups to their presumed parental species. We performed an additional phylogenetic reconstruction, including hybrid populations to confirm the phylogenetic placement of these putative hybrids. We started by quality-filtering the raw Illumina data by identifying and removing adapters and low-quality basepairs using *AdapterRemoval v2.3.2* [42]. Subsequently, we used *Skmer v3.2.1*, a pipeline designed to estimate genetic distances between genomes through a *k*-mer-based approach. *Skmer* offers an approach for phylogenetic reconstruction without requiring an assembly and accounts for low-coverage of the samples [43–46]. Running *Skmer* involved running the module ‘skmer reference’ to process the adapter-trimmed sequencing data, followed by ‘skmer subsample’ to create 100 subsamples of the libraries, and ‘skmer correct’ to correct the distance matrices based on these subsample replicates. To construct a distance-based phylogenetic tree we employed *fastme v2.1.5* [47].

### TE library construction

We downloaded the reference genome of the sunflower (*Helianthus annuus*) from its official website (https://www.sunflowergenome.org/; version Ha412HOv2.0-20181130), and used it to generate a sunflower-based TE and repeat library, with the intention of classifying repeats in *Scalesia* and *Pappobolus*, using *Repeat Modeler v2.0.2* [48]. The Sunflower genome was selected as a reference because it has a high-quality reference genome, and it is equally evolutionary distant from *Scalesia* and *Pappobolus* [49]. Building a TE and repeat library based on *Scalesia* alone could introduce biases when conducting comparisons with the outgroup taxa (but see below). We used the sunflower-based as the base library for subsequent TE detection using *dnaPipeTE*.

We used *dnaPipeTE* [50] to extract and characterize TEs and repeat sequences. *dnaPipeTE* leverages the RNA-seq assembly pipeline Trinity to extract, quantify and assemble sequences, including TEs and other repeats. While the primary objective of *dnaPipeTE* is to construct representative repeat library, it also detects and quantifies partially assembled TE and repeats (which suffice for estimating the relative abundances on which we rely in this study). Upon the completion of the assembly and collection process, *dnaPipeTE* categorises TEs and repeats into different classes based on their homology with the TE and repeat library. In the established sunflower library, TE classes included DNA, Helitron, Long interspersed nuclear element (LINE), Long terminal reads (LTR), rRNA, and Short interspersed nuclear elements (SINE). Leveraging *RepeatMasker*, *dnaPipeTE* identifies low-complexity sequences, simple repeats, and satellites, guided by a specific coverage threshold. *dnaPipeTE* has been successfully applied to a diverse array of species, including arthropods, mollusks, and vertebrates (reviewed in [51]), even revealing substantial variation in TE and repeat content at the population-level (e.g [52]). *dnaPipeTE* is designed to work low-coverage data (< 1×), identifying TEs and repeats by assembling contigs with high coverage (indicating a high copy number within the genome) and subsequently using a database to classify these genomic structures. To check whether lower coverages would not influence our results, we simulated reads using ART [53], specifying Illumina data, 150 bp read length, 10× coverage, and an insert size of 500 bp with a standard deviation of 50 bp. We simulated reads for three randomly selected *Scalesia* chromosomes (12, 19 and 23) and ran *dnaPipeTE* specifying the size of the chromosome and different coverages: (0.1×, 0.2×, 0.5×, 1×, 2×, 5×), three times for each chromosome. This showed that the major classes of TEs/repeats were quite similar (Supplementary Fig. 1). We then ran *dnaPipeTE* on single-end forward reads for each individual separately, downscaling the data to 0.5× coverage, specifying a genome size of 3.2 Gbp [39], one Trinity assembly iteration, and a minimal query percentage of 0.2 [50].

### Exploration of patterns

We extracted three outputs from *dnaPipeTE*: (1) counts of TEs and repeats, which allowed a direct comparison of the number of TEs and repeats accumulated across species; (2) repeat landscape plots, which offered insights into the accumulation of TEs through time (to complement this data, we also ran a repeat accumulation analysis in the chromosome-resolved *Scalesia atractyloides* genome assembly [39] using RepeatMasker. We used the Kimura substitution level for repeat landscape plots as an indirect metric of TE divergence. This approach is based on the Kimura two-parameter model, which accounts for the differences in transition and transversion rates during nucleotide substitution. This metric is often used as a proxy for the age of TEs, with higher Kimura values indicating older, more diverged elements. While this approach is widely used in TE studies due to its simplicity and computational efficiency, it does have limitations. For example, it assumes a constant rate of evolution across sites and lineages, which may not always hold true; and 3) fasta files containing TE and repeat sequences. Using the sequences in a fasta format, we used *OrthoFinder* to group TEs and repeats based on sequence similarity [54, 55], and examined the number of TE/repeat orthogroups shared between the outgroup species and *Scalesia* using *UpSet plots* and *Venn diagrams*. To test for differences between groups, we performed a Mann–Whitney U test comparing hybrids vs. non-hybrids, and humid (*S. cordata*,* S. microcephala*,* S. pedunculata*) vs. arid (*S. villosa*,* S. stewartii*,* S. atractyloides*,* S. crockeri*,* S. aspera*,* S. retroflexa*,* S. helleri*,* S. gordilloi*,* S. incisa*,* S. divisa*).

We considered the possibility that the limited variation in TE and repeat content observed in some of our results was due to the use of a sunflower-based library, which might be too divergent from *Scalesia* and *Pappobolus*. To address this concern, we repeated all the aforementioned analyses using a *Scalesia*-specific library instead of the sunflower library. To obtain the *Scalesia*-specific library, we ran *RepeatModeler* on the *Scalesia atractyloides* genome [39] and ran *DNApipeTE* analyses using the same parameters, and running analyses involving the exploration of TE and repeats). Analyses of the *Scalesia*-specific library found similar patterns to the main results (Supplementary Fig. 2).

## Results

The phylogenetic tree is comprised mostly of short internal branches in *Scalesia*, as expected in a rapid radiation. *Scalesia villosa* is positioned as the sister species to all the remaining *Scalesia* species (Fig. 1A; Supplementary Fig. 3). *S. stewartii* and *S. atractyloides* form a clade which is sister to the remaining 12 species. *S. affinis*, *S. microcephala*, and *S. cordata* are grouped together as a clade being sister to a clade including nine *Scalesia* species, namely *S. crockeri*, *S. aspera*, *S. retroflexa*, *S. helleri*, *S. baurii*, *S. pedunculata*, *S. gordilloi*, *S. incisa*, and *S. divisa* (Fig. 1A; Supplementary Fig. 3). The phylogenetic analysis including hybrid populations placed all hybrids as sister to one of the parental species (Supplementary Fig. 4).

We observed little variation in TE counts among different species, both in relative and absolute terms (Fig. 1B; Supplementary Table 2). When considering all TE classes classified by *DnaPipeTE* (LTR, LINE, SINE, DNA, Helitron, rRNA, Low_Complexity, Satellite, Simple_repeat, others, na), the outgroup lineages displayed a higher count of TEs and repeats, with an average of 1,382,957,707 bp (max 1‚398,040,697 bp, min 1,368,357,206 bp, standard deviation 12,799,060 bp). *Scalesia* species (excluding hybrids) exhibited a lower TE and repeat content, with an average of 1,249,051,887 bp (max 1,264,569,945 bp, min 1,223,889,466 bp, standard deviation 1,1922,527 bp). Hybrid *Scalesia* populations displayed a TE and repeat content averaging 1,253,765,218 bp (max 1,265,750,267 bp, min 1,239,589,542 bp, standard deviation 12,633,031 bp; Fig. 1B). Both Mann-Whitney U tests were non-significant (humid vs. arid: W = 16, *P* = 1; hybrid vs. non-hybrid; W = 41, *P* = 0.5744).

Long terminal repeat (LTR) retrotransposons were the largest class of TEs found in the data. *Scalesia* species (hybrids excluded) had an average of 518,734,809 bp of their genomes composed of LTRs (max 542,466,614 bp, min 439,269,606 bp, standard deviation 24,787,195 bp). The outgroup species had a mean of 719,315,917 bp of their genomes composed of LTRs (max 755,592,383 bp, min 693,803,469 bp, standard deviation 21,926,958 bp), while hybrid *Scalesia* populations had 532,048,025 bp of LTRs (max 553,999,518 bp, min 504,436,424 bp, standard deviation 23,478,054 bp). In the second largest class of TEs, DNA elements, *Scalesia* had an average of 53,397,161 bp composed of DNA elements (max 56,653,585 bp, min 45,126,916 bp, standard deviation 2,748,119 bp), while hybrid populations had 53,374,532 bp (max 54,154,091 bp, min 51,526,434 bp, standard deviation 1,238,711 bp). In contrast, the outgroup exhibited fewer DNA elements, with an average of 32,145,458 bp (max 34,376,438 bp, min 30,424,080 bp, standard deviation 1,414,881 bp).

### Repeat landscape plots

**Fig. 2:**
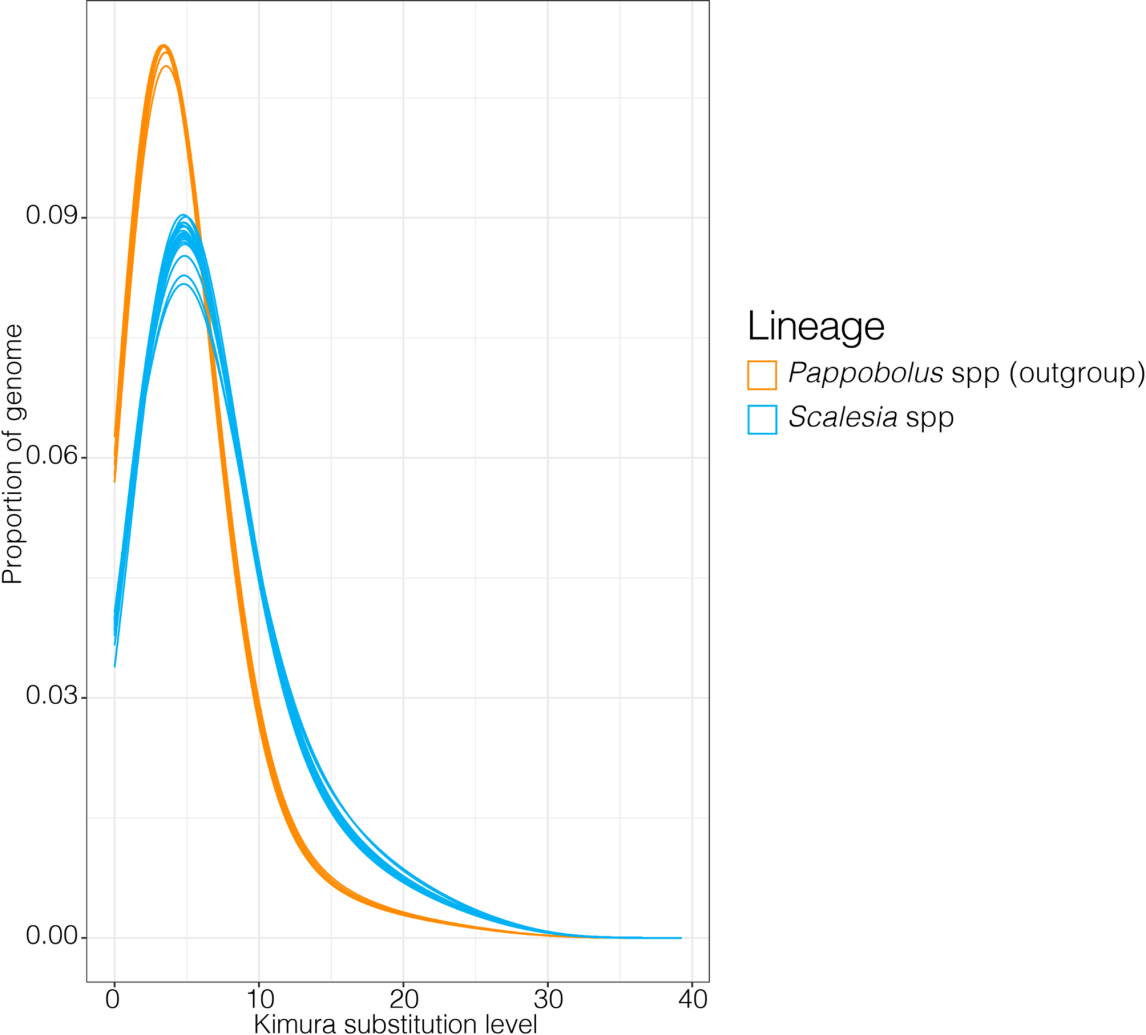
Repeat/transposable element landscape plot. The *x-axis* shows the Kimura substitution level as a metric to estimate the divergence of transposable elements, the *y-axis* shows the portion of the genome covered by TEs and repeats. Assuming a constant mutation rate, higher Kimura substitution levels indicate older TE ages. The outgroup (genus *Pappobolus*) is plotted in orange, and all *Scalesia* species in blue. Repeat accumulation landscape plots for each specimen, coloured by TE classes, are on Supplementary Figs. 7–11

The repeat landscape plot shows that all *Scalesia* species exhibit a similar pattern of TE and repeat accumulation (Fig. 2; Supplementary Fig. 5). All outgroup species have a similar accumulation of TEs, which is distinct from that of *Scalesia*. Specifically, there is a younger accumulation of TEs in the outgroup relative to *Scalesia* (Fig. 2; Supplementary Fig. 5). In all the landscape plots, the TE/repeat accumulation peak of *Scalesia* is at a Kimura substitution level of approximately 5 (Fig. 2; Supplementary Figs. 6). The repeat landscape plots decomposed in different TE and repeat classes show that the accumulation of TEs and repeats does not differ when comparing the outgroup (Supplementary Fig. 7), *Scalesia* (Supplementary Fig. 8–10), and *Scalesia* hybrids (Supplementary Fig. 11).

### Organization of genome-specific elements

**Fig. 3:**
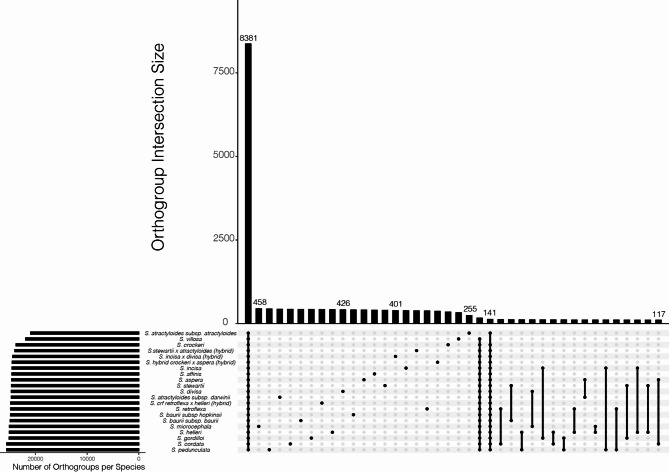
UpSet plot of long terminal repeat elements (LTR) identified within the *Scalesia* radiation (other TE and repeat groups are found in Supplementary Figs. 12–16). The *UpSet* plot is an alternative visualisation to a Venn diagram where the y-axis on the top graphic shows a frequency of LTR orthogroups (groups of closely related LTRs) and the x-axis shows different combinations of the data (the first column includes orthogroups present in all *Scalesia*, the second column are orthogroups exclusive to S. *retroflexa x helleri* hybrids, the third column are orthogroups exclusive to *S. atractyloides*). Dark grey circles highlight species combinations

Using *OrthoFinder*, we grouped TEs and repeats into orthogroups, that are sets of orthologous sequences derived from the last common ancestor of all species under consideration (Fig. 2; Supplementary Figs. 12–16). We found that the majority of TE orthogroups are present in all *Scalesia*. The sole exception was in the satellite elements, for which we detected very few shared orthologs. In the largest class, LTR, we found 8,381 orthogroups present in all *Scalesia* genomes, while only 458 − 255 orthogroups were found to be unique to a single genome (Fig. 3).

Superfamily-level analyses show that TE figures were remarkably even in *Scalesia* (Table 1) and the outgroup (Table 1). With the exception of LTR/Gypsy and LTR/Copia, all TE counts were below 50,000 elements and were remarkably even across *Scalesia* species. LTR/Gypsy and LTR/Copia included between 2,500,000–983,398 elements (Table 1).

**Fig. 4:**
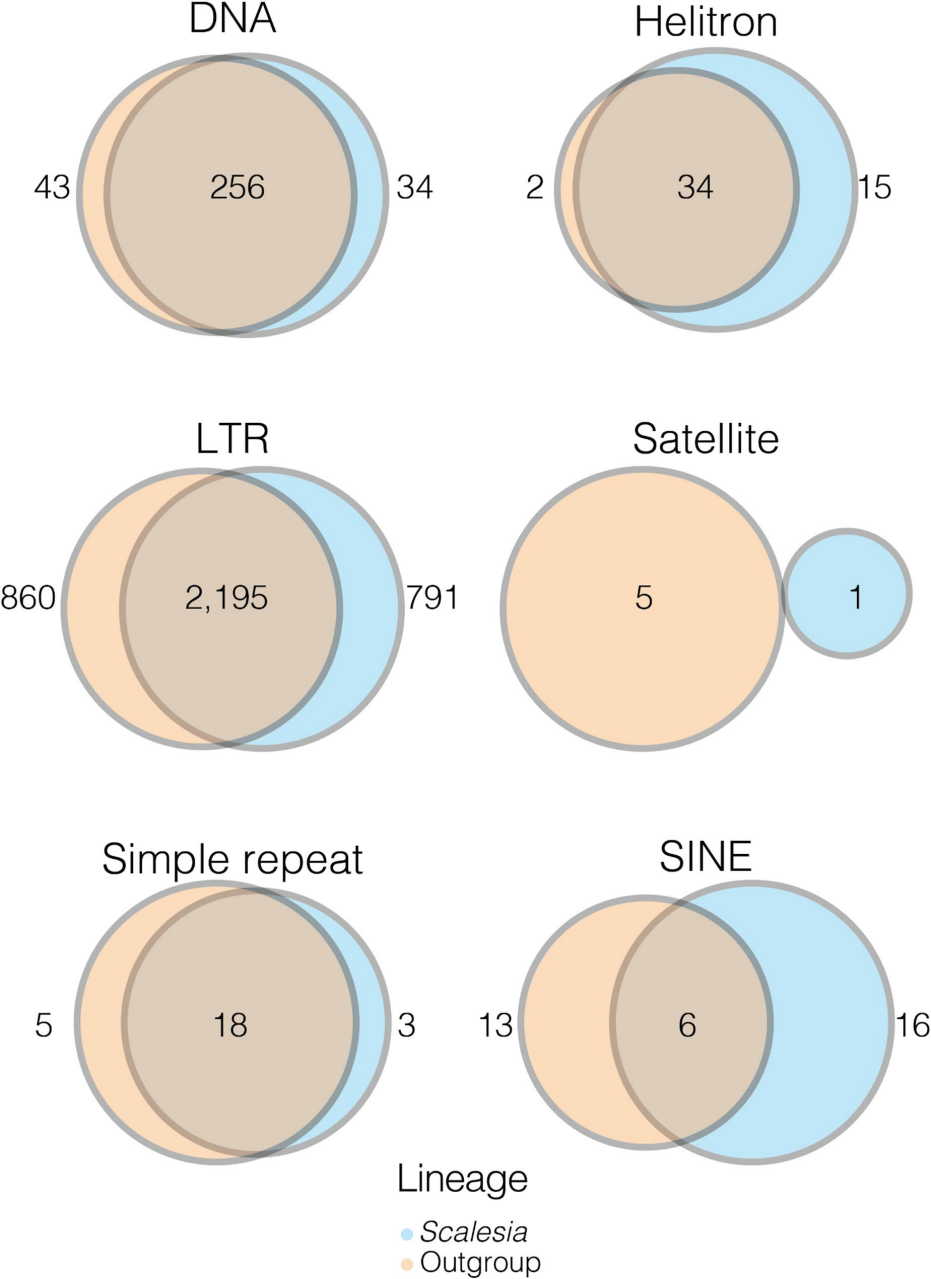
Representation of orthogroups present in *Scalesia* (blue) or the outgroup (orange). Shared orthogroups are given as overlap for different TEs and repeats

We used *OrthoFinder* to identify orthogroups of TEs and repeats in five randomly chosen *Scalesia* species and five outgroup *Pappobolus* species, and parsed the results to compare counts of *Scalesia*-specific orthogroups, outgroup-specific orthogroups, and orthogroups shared between the two genera (Fig. 4). We chose to downscale the data to five random species because we observed imbalances in estimations that arose from analysing the 15 *Scalesia* species in comparison to only five outgroup species (two specimens of *P. hypargyreus* were included; Figure 4). Since we downscaled the data to only five *Scalesia* species, we repeated the analyses three times, finding that the results were consistent across the three iterations (Supplementary Table 3). The largest three classes of TEs (LTR, DNA and Helitron) had a majority of its orthogroups common to *Scalesia* and the outgroup. *Scalesia* had more private SINEs than the outgroup (Fig. 4).

**Table 1:** TE superfamily counts for different *Scalesia* species

	SINE/ RTE	SINE/ tRNA	RC/ Helitron	LTR/ Pao	LTR/ Gypsy	LTR/ ERVK	LTR/ Copia	LTR/ Caulimovirus	LTR/ Cassandra	LTR	LINE/ RTE	LINE/ R1	LINE/ L2	LINE/ L1	LINE/ CRE	DNA/ hAT	DNA/ TcMar	DNA/ PIF	DNA/ MULE	DNA/ CMC
*P. ecuadoriensis*	371	3869	16,350	371	3,305,984	5692	983,123	4440	4347	4602	2868	1323	2357	39,398	6894	58,136	4195	31,012	41,568	32,994
*P. hypargyreus (a34)*	507	3137	16,022	655	3,462,121	6185	1,106,014	3313	9154	5193	3262	1049	9222	45,614	6640	56,465	4514	33,856	46,674	34,807
*P. hypargyreus (a62)*	490	3327	17,824	579	3,245,817	5647	1,031,933	3133	5205	4991	2962	1197	4796	38,229	7595	60,699	4821	31,621	42,205	35,443
*P. juncosae*	489	3623	16,661	693	3,204,955	6596	984,296	2785	5088	5405	3091	1152	6176	41,903	6190	59,839	5139	32,296	43,978	36,171
*P. lehmanii*	463	3303	22,877	725	3,303,580	7383	1,087,988	2421	4651	5866	3257	1739	8888	42,572	6177	56,389	4068	32,610	47,563	39,326
*P. nigrescens*	421	3642	21,975	847	3,271,270	7511	1,130,551	1772	7352	5421	3635	1885	6893	43,556	6718	60,297	3801	33,479	50,037	39,566
*S. affinis*	1567	10,155	12,724	1531	2,062,556	1841	1,117,210	10,569	10,179	2327	7858	3577	1606	51,784	19,981	127,256	4571	33,466	59,219	46,262
*S. aspera*	1538	10,260	12,622	1392	2,054,923	1766	1,130,845	9470	9795	2681	8171	3713	1052	48,721	21,387	128,320	4706	33,549	56,738	45,085
*S. atractyloides subsp. atractyloides*	1490	11,041	11,366	1604	1,903,215	1395	1,075,429	11,533	6980	2431	8083	3499	1046	46,974	19,831	121,523	4734	30,840	54,040	48,147
*S. atractyloides subsp. darwinii*	1767	9377	12,187	1606	1,934,233	1671	1,117,869	10,582	8163	2168	8081	3386	1175	49,308	20,594	130,617	5088	35,540	59,713	53,550
*S. baurii subsp. baurii*	1679	9749	11,530	1576	1,966,602	1587	1,135,162	7296	11,271	2086	7809	4015	1179	48,009	18,545	130,197	4874	33,608	58,129	49,340
*S. baurii subsp. hopkinsii*	1799	9588	15,327	1239	1,971,288	1198	1,161,059	8856	8890	2307	7955	3936	1441	49,700	19,138	129,198	5052	32,873	54,939	49,547
*S. cordata*	1932	10,611	13,617	1756	1,954,952	1061	1,129,924	7630	8952	2888	8293	2961	1793	44,308	20,436	129,685	4999	34,620	59,119	48,519
*S. crockeri*	1519	10,132	14,243	1605	2,010,330	1020	1,126,152	8133	7828	2359	7711	1968	1342	48,434	20,218	129,001	4698	33,944	60,177	49,633
*S. divisa*	1743	11,060	11,802	1675	1,968,864	1852	1,125,339	8803	10,585	2762	7818	3033	1310	47,924	20,110	134,834	6359	34,272	58,448	47,176
*S. gordilloi*	1995	9419	12,625	1688	1,862,603	1314	1,104,228	9551	7757	1930	7933	3512	1134	46,699	17,953	128,123	5017	32,840	56,922	50,052
*S. helleri*	1643	10,275	11,464	1659	1,910,324	834	1,134,235	8620	6981	2212	8186	4566	1166	55,736	18,181	131,455	5307	33,705	61,172	50,961
*S. crf retroflexa x helleri (hybrid)*	1961	10,879	12,201	1643	1,976,368	1093	1,120,134	7676	7716	2411	8120	3053	1375	51,217	20,382	129,192	4911	35,959	59,197	47,648
*S. crockeri x aspera (hybrid)*	1556	10,280	12,973	1551	1,887,477	1822	1,092,086	10,715	8552	2241	7536	3197	1757	45,653	18,382	124,401	4924	31,965	55,807	46,250
*S. incisa x divisa (hybrid)*	1334	10,138	13,626	1670	2,074,431	1482	1,171,670	8133	9377	2444	7914	4062	1121	52,294	20,908	126,123	4947	34,492	60,656	50,053
*S. stewartii x atractyloides (hybrid)*	1907	10,495	10,714	1456	2,116,783	1891	1,142,462	8939	11,942	2764	7777	2604	1067	51,784	21,833	124,489	4737	34,289	64,957	47,572
*S. incisa*	1833	9810	12,080	1375	1,965,781	1199	1,147,144	9086	9953	2245	7687	3302	1923	47,576	18,695	126,281	4535	32,910	55,614	46,353
*S. microcephala*	1536	10,066	13,042	1830	2,023,813	1232	1,174,987	10,284	9920	2387	9155	4019	1300	47,641	20,498	129,912	5066	34,970	66,414	54,403
*S. pedunculata*	1905	9834	14,041	1596	1,798,210	910	1,081,409	9475	7362	1886	8369	3102	1318	48,246	17,922	133,988	4951	32,767	53,913	45,367
*S. retroflexa*	2062	10,596	11,001	1958	2,007,466	1521	1,144,562	7991	8248	2378	8331	4019	1304	49,004	21,464	125,144	5273	34,879	63,426	49,993
*S. stewartii*	1884	10,133	11,476	1831	1,990,804	2700	1,100,148	9441	12,371	2646	8339	3255	1497	56,637	21,333	130,720	4795	32,633	70,144	47,267
*S. villosa*	1457	7631	11,937	1176	1,626,877	1630	983,398	10,836	5610	1643	6482	3192	869	43,475	14,025	116,518	4492	26,792	45,928	41,713

## Discussion

Transposable elements (TEs) play a crucial role in shaping genomes and in spurring phenotype diversity. Here, we tested whether rapid diversification, shifts in climatic niches or hybridization are associated with an increase of TE accumulation in the genomes of the genus *Scalesia*, a radiation endemic to the Galápagos (Fig. 1). Our results show that TE or repeat accumulation is consistent across the *Scalesia* phylogeny, with no major changes across species (Fig. 1) or over time (Fig. 2). We also found no evidence of lineage-specific TE or repeat expansions, as most TE groups were found across all species (Fig. 3), in similar numbers across the explored genomes (Table 1), and shared with the outgroup (Fig. 4). Our findings indicate that neither rapid diversification, hybridization, nor ecological niche shifts are significantly associated with TE and repeat content variation within the *Scalesia* adaptive radiation. This suggests that greater nuance is needed in the literature regarding the role of TEs: while they can be crucial at the locus level, driving the evolution of novel phenotypes, their macroevolutionary correlations between TE content and diversification or speciation appear tenuous.

### Limited variation in TE content in *Scalesia* despite rapid diversification

We found no major differences in TE or repeat content in different *Scalesia* species in any of the analyses performed and focusing on phylogenetic comparisons, accumulation over time, and class-specific expansions (Figs. 1–05). The lack of variation may be attributable to the methods used, but this seems unlikely since *dnaPipeTE* retrieved variation between *Pappobolus* and *Scalesia*, suggesting that the two lineages have accumulated different TEs and repeats after their split, about 3 million years ago [29]. *dnaPipeTE* has been shown to detect population-level differences [52, 56–58], and between closely related species across the Tree of Life [59–65]. One tempting explanation for the lack of variation is that *dnaPipeTE* may be biased to find conserved old elements, thereby explaining the observed patterns. However, this is not the case as *dnaPipeTE* is very sensitive in detecting recent TE families, being less sensitive to older TE families due to increased divergence and short reads that limits alignments, as shown for the human genome (see supplementary data included in [50]). We therefore suggest that TEs are under phylogenetic inertia, as shown for various *Drosophila* species [66]. Our findings suggest that the fundamental TE composition remains conserved across *Scalesia* lineages.

The lack of variation in TE and repeat content in *Scalesia* is noteworthy. In our dataset, we observe a variation of 4.60% in LTRs, and 4.58% of variation in DNA elements. This variation is very little when compared to other works focusing on congeneric species. For instance, in the plant genus *Passifora*, Tekay LTR-elements varied between 8.52 and 35.71% and Angela LTR-elements between 1.96 and 29.05% (absolute estimates considering the whole genome; [67]. In oaks (*Quercus*) LTRs varied between 34.07 and 55.05% (relative TE content; [68]. In *Rhodnius* bugs, with different ecologies, Tc1/Mariner elements varied between 14.3 and 25% [65], while in *Diabrotica* beetles MITE elements varied between 150 and 610 MegaBasepairs [64].

One potential explanation for the observed lack of species-specific TEs is the high repeat content of the *Scalesia* genome. Our previous analyses revealed that approximately 80% of the *Scalesia atractyloides* genome is composed of repetitive sequences [39]. This high repeat content could be due to the presence of numerous repeat families that actively replicate with a strong phylogenetic signal, rather than a dynamic turnover of species-specific repeat families with a low copy number. In this regard, Novák and colleagues (2020) found that TE content reaches a plateau at around 80% across angiosperms with genome sizes between 5 and 10 Gbp [69]. It is possible that a plateau of TE content could be maintained, with few families represented at high numbers, and that a dynamic turnover occurs within families, rather than between TE families.

Our findings challenge the notion that lineages with high diversification and speciation rates also display elevated rates of TE content accumulation. One caveat to this interpretation is that the sister lineage to *Scalesia*, including *Pappobolus*, may have undergone similar diversification on the mainland [29]. In any case, the role of TEs in adaptive radiation remains debated, with some studies demonstrating their importance as drivers of radiation [70, 71], while others proposing a less significant role [27, 28]. This discrepancy likely stems from the scale and data employed. Specifically, the correlation between TE content and diversification is mostly based on limited genomic data, correlational analyses and a narrow taxonomic scope, and this might have led to an overestimation of TEs’ role. For instance, an initial analysis of four transcriptomes and genomes of East African cichlids, compared to tilapia and other teleosts, suggested a higher accumulation of TE counts during the radiation [26]. In contrast, recent evidence involving analyses of hundreds of genomes found no relationship between species diversity and TEs within different tribes of the Tanganyika radiation [27]. While this does not negate the compelling experimental evidence supporting the role of TEs in driving the evolution of specific phenotypic change, such as the elegant findings of Kratochwil and colleagues demonstrating that a TE insertion triggered the emergence of a novel phenotype, the cichlid gold morph [11], it casts doubt on a macroevolutionary role of TEs in diversification dynamics. Our data, which spans an entire adaptive radiation, seems to support the notion that TE accumulation and diversification are not correlated. Considering this, we suggest that caution should be taken with potential overestimation of the role of TEs as major driving forces in speciation and ecological diversification at the macroevolutionary scale in all cases (e.g [15–22, 72–74]). While TEs offer a convenient explanation for rapid evolution, it is likely that they are merely “along for the ride” and may only be purged when they reach problematic levels of replication.

### TEs and ecological variation

The minimal variation in TE accumulation observed in *Scalesia* suggests that the climatic niche does not significantly influence TE content within the radiation. In adaptive radiations, closely related species typically occupy distinct ecological niches [24, 25, 75]. In *Scalesia*, species exhibit distinct climatic niches (Fig. 1) [29, 30, 40], with temperature and the presence of arid environments being key factors shaping the evolution of the radiation [29, 39, 40]. Various studies have demonstrated that aridity exerts a significant selective pressure on plant genome size reduction (e.g [14, 76])., as this correlates nearly 1-to-1 with smaller cell sizes [77]. Smaller cells improve water economy as smaller stomata respond more efficiently to water stress, enhance gas exchange regulation, and optimize photosynthetic performance [31, 78, 79]. Given that arid environments favor smaller cells, aridity is hypothesized to be an important selective pressure regulating genome size [14]. Therefore, it would be expected that TEs are being purged in species occupying arid coastal areas (e.g. *S. affinis*), where temperatures frequently reach 40 °C throughout the year and water stress is pervasive, compared to species found in the humid highlands (e.g., *S. pedunculata*), where conditions are consistently moist and cooler. This leads to an alternative hypothesis: selection is unable to effectively remove TE as the removal of a single TE, only a few kilobases in size, might be ‘invisible’ in a genome of 3–4 gigabases [80].

### TEs and hybridization

The four putative hybrid populations, diagnosed based on intermediate morphology, examined in this study exhibited similar TE content to non-hybrid populations, excluding the possibility of TE burst as a result of hybridization. Traditionally, hybridization has been perceived as a detrimental force to the integrity of species [81], and it has been proposed that hybridization may lead to genome destabilisation by promoting TE activity [1, 82]. The presence of more TEs in hybrid populations would be expected in a scenario involving TE bursts triggered by multi-generational hybridization. In such cases, increased TE transcription should lead to more TEs insertions in the hybrid genomes, and a consequent increase of genome size. However, the case in *Scalesia*, as evidenced by the absence of supporting patterns in the repeat accumulation curves (Fig. 2), the lack of superfamily expansion or super-family level (Fig. 3), and absolute accumulation (Fig. 1). These findings are in agreement with those of [83], who demonstrated no evidence of LTR retrotransposon proliferation when comparing multiple hybrid populations of *Helianthus annuus* (sunflower) × *H. petiolaris.* This result suggests that post-transcriptional repression mechanisms may prevent the proliferation of LTRs [83]. A similar scenario may be at play in *Scalesia*, but additional data would be required to confirm this hypothesis.

## Conclusions

Our results highlight the importance of distinguishing between the locus-specific effects of TEs and their global genomic impact (‘bursts of TEs’). Transposable elements are undeniably important drivers of locus-specific and phenotypic-specific evolution, as evidenced by numerous studies demonstrating their role in generating novel genetic variation and facilitating adaptive traits [9, 11]. However, our findings in the *Scalesia* radiation challenge the notion that global bursts of TE activity are a primary driver of diversification at macroevolutionary scales. Despite rapid speciation, ecological niche shifts, and hybridization events within Scalesia, we observed remarkably stable TE accumulation across species, with no significant variation linked to diversification, climatic adaptation, or hybridization. This suggests that while TEs may play a critical role in generating localized genetic variation and phenotypic innovation, their broader impact on lineage-wide diversification and adaptive radiation may be overstated.

## Electronic supplementary material

Below is the link to the electronic supplementary material.

Supplementary Material 1

## Data Availability

The raw sequencing data generated for this study can be found at the European Nucleotide Archive under accession code PRJEB70770.
