# Supplementary material for: No evidence of transposable element bursts in the Galápagos Scalesia adaptive radiation despite hybridization, diversification and ecological niche shifts
Source: Mob DNA. 2025 May 31;16:23. doi: 10.1186/s13100-025-00362-z (PMC12125827; doi:10.1186/s13100-025-00362-z)

**Supplementary Table 01** Specimens used with sampling coordinates and information. Specimen CDRS 17571 was collected by Walter Simbaña.

| **Species** | **Individual identifier** | **Sampling location** | **Island** | **Collection date** |
| --- | --- | --- | --- | --- |
| *Scalesia villosa* | HAMP 24_22 | 1° 13' 55.0"S  90° 21' 41.0"W | Floreana | 1998-01-20 |
| *S. stewartii* | HALM 36_6 | 0° 17' 21.9'' S  90° 34' 27.4'' W | Santiago | 1999-02-03 |
| *S. atractyloides var. darwinii* | CDRS 17604 | 0° 17' 52.8966" S 90° 38' 31.6968" W | Santiago | 2005-07-18 |
| *S. atractyloides* | CDRS 16393 | 0° 18' 27.8958" S 90° 37' 17.9976" W | Santiago | 2004-02-26 |
| *S. affinis* | HALM 48_21 | 0° 55' 18.3''S  90° 59' 18.5''W | Isabela | 1999-02-11 |
| *S. microcephala* | HALM 51_23 | (Darwin Volcano) | Isabela | 1999-02-06 |
| *S. cordata* | HALM 44_18 | 0° 50' 48.1'' S  91° 01' 18.9'' W | Isabela | 1999-02-10 |
| *S. crockeri* | HALM 39_23 | 0° 34' 45.1'' S  90° 10' 22.8'' W | Santa Cruz | 1999-02-06 |
| *S. aspera* | HALM 37_13 | 0° 33' 36.3'' S  90° 31' 59.9'' W | Floreana | 1999-02-03 |
| *S. retroflexa* | HALM 26_22 | 0° 44' 40.7'' S  90° 16' 22.1'' W | Santa Cruz | 1999-01-26 |
| *S. helleri* | HALM 62_14 | 0° 45' 15.5'' S  90° 25' 26.8'' W | Santa Cruz | 1999-05-24 |
| *S. baurii subsp. hopkinsii* | SLAM 20_17 | 0° 31' 51.1'' N  90° 44' 19.6'' W | Pinta | 2000-01-11 |
| *S. baurii subsp. baurii* | AHA 6_15 | 0° 36' 23'' S  90° 40' 12'' W | Pinzon | 2004-02-16 |
| *S. pedunculata* | HALM 25_1 | 0° 37' 27'' S  90° 23' 02'' W | Santa Cruz | 1999-01-25 |
| *S. gordilloi* | HAMP 38_18 | 0° 55' 49'' S  89° 36' 29'' W | San Cristóbal | 1998-01-26 |
| *S. incisa* | HALM 20_28 | 0° 41' 44.4'' S  89° 18' 03.4'' W | San Cristóbal | 1999-01-21 |
| *S. divisa* | HALM 11_8 | 0° 47' 26.2'' S  89° 20' 07.7'' W | San Cristóbal | 1999-01-17 |
| *S. retroflexa x helleri (hybrid)* | HALM 41_22 | 0° 41' 40.1'' S  90° 11' 28.2'' W | Santa Cruz | 1999-02-06 |
| *S. crockeri x aspera (hybrid)* | HALM 8_22 | 0° 32' 21.2'' S  90° 19' 01.6'' W | Santa Cruz | 1999-01-15 |
| *S. incisa x divisa (hybrid)* | HALM 23_26 | 0° 49' 07'' S 89° 21’ 12’’ W | San Cristóbal | 1999-01-13 |
| *S. stewartii x atractyloides (hybrid)* | CDRS 17571 | - | Santiago | 2004-07-31 |

**Supplementary Table 02** The total number of base pairs encompassed within the genomes of various species by distinct TEs and repeats, as determined using DNApipeTE. All units are given in base pairs. Na = Not available (TE/repeat not classified by the pipeline)

| **Species** | **DNA** | **Helitron** | **LINE** | **Low Complexity** | **LTR** | **Na** | **rRNA** | **Satellite** | **Simple repeat** | **SINE** | **others** |
| --- | --- | --- | --- | --- | --- | --- | --- | --- | --- | --- | --- |
| *Scalesia villosa* | 45,126,916 | 1,946,883 | 10,385,579 | 9,133,225 | 439,269,606 | 322,271,738 | 3,008,674 | 102,163 | 29,655,171 | 1,456,493 | 387,282,505 |
| *S. stewartii* | 55,786,000 | 1,993,293 | 14,006,588 | 9,948,179 | 526,075,924 | 186,059,625 | 193,344 | 278,117 | 26,689,223 | 1,660,095 | 432,157,203 |
| *S. atractyloides subs. darwinii* | 56,653,585 | 2,041,560 | 12,441,394 | 8,950,844 | 518,269,106 | 171,152,155 | 1,949,844 | 192,828 | 28,458,279 | 1,392,701 | 447,888,951 |
| *S. atractyloides subsp. atractyloides* | 50,822,329 | 1,810,573 | 12,096,200 | 7,650,064 | 499,904,799 | 190,980,264 | 4,741,531 | 114,753 | 29,365,260 | 1,562,290 | 424,841,403 |
| *S. affinis* | 54,094,009 | 2,091,180 | 12,652,563 | 7,519,028 | 537,218,659 | 173,899,049 | 4,054,658 | 197,597 | 33,114,524 | 1,469,608 | 428,321,568 |
| *S. microcephala* | 55,378,305 | 2,229,856 | 12,203,958 | 8,904,756 | 542,466,614 | 162,802,621 | 8,096,237 | 160,830 | 30,424,327 | 1,432,401 | 438,798,946 |
| *S. cordata* | 53,050,651 | 2,238,390 | 11,480,001 | 8,105,577 | 521,467,514 | 181,531,733 | 2,973,111 | 127,466 | 29,020,614 | 1,594,033 | 435,422,629 |
| *S. crockeri* | 53,221,973 | 2,211,414 | 11,395,625 | 6,052,970 | 535,505,912 | 149,972,737 | 2,880,740 | 268,332 | 29,268,255 | 1,470,532 | 436,740,190 |
| *S. aspera* | 52,203,387 | 1,976,223 | 12,331,201 | 7,909,834 | 540,267,886 | 184,448,121 | 6,202,336 | 241,711 | 27,674,376 | 1,464,590 | 429,850,280 |
| *S. retroflexa* | 55,251,736 | 1,837,107 | 12,476,917 | 7,699,004 | 536,733,186 | 171,059,766 | 1,949,251 | 144,491 | 29,643,549 | 1,580,131 | 442,444,569 |
| *S. helleri* | 56,020,313 | 2,046,192 | 13,383,026 | 10,308,193 | 516,661,345 | 177,487,938 | 3,935,280 | 577,311 | 26,126,213 | 1,515,017 | 435,911,784 |
| *S. baurii subsp. hopkinsii* | 52,716,511 | 2,399,494 | 12,573,365 | 7,785,674 | 526,528,686 | 175,858,294 | 2,749,669 | 215,142 | 31,013,132 | 1,445,155 | 440,791,295 |
| *S. baurii subsp. baurii* | 55,082,960 | 2,081,142 | 12,565,754 | 9,368,438 | 525,703,451 | 169,123,674 | 4,297,930 | 152,429 | 28,723,975 | 1,413,881 | 441,798,333 |
| *S. pedunculata* | 52,898,068 | 2,281,302 | 12,211,247 | 9,206,590 | 492,275,095 | 194,149,470 | 2,357,555 | 160,667 | 28,690,578 | 1,467,974 | 436,207,976 |
| *S. gordilloi* | 55,248,501 | 2,227,922 | 11,215,092 | 9,104,374 | 506,746,761 | 195,614,820 | 3,661,194 | 326,646 | 29,118,472 | 1,486,833 | 426,464,882 |
| *S. incisa* | 50,907,853 | 2,065,405 | 11,676,042 | 8,017,341 | 531,259,324 | 198,239,407 | 4,842,390 | 224,166 | 28,568,687 | 1,455,124 | 424,955,998 |
| *S. divisa* | 53,288,635 | 2,146,792 | 11,904,225 | 9,345,728 | 522,137,887 | 180,739,120 | 2,408,766 | 271,511 | 29,840,205 | 1,599,547 | 439,961,049 |
| *S. retroflexa x helleri (hybrid)* | 53,976,414 | 2,140,621 | 13,138,449 | 7,304,080 | 520,853,635 | 182,091,443 | 2,575,867 | 153,366 | 28,275,814 | 1,623,087 | 434,610,189 |
| *S. crockeri x aspera (hybrid)* | 51,526,434 | 2,159,025 | 11,711,264 | 7,232,581 | 504,436,424 | 197,665,137 | 3,681,341 | 167,443 | 29,199,152 | 1,429,044 | 430,381,697 |
| *S. incisa x divisa (hybrid)* | 54,154,091 | 2,255,980 | 12,809,498 | 6,989,578 | 548,902,524 | 164,349,221 | 4,432,734 | 227,643 | 31,939,589 | 1,553,339 | 438,136,070 |
| *S. stewartii x atractyloides (hybrid)* | 53,841,192 | 1,854,685 | 12,737,379 | 8,076,603 | 553,999,518 | 176,028,156 | 2,607,345 | 121,790 | 27,285,298 | 1,586,497 | 424,949,246 |
| *P. ecuadoriensis (outgroup)* | 30,424,080 | 3,309,404 | 8,527,180 | 2,541,483 | 707,567,653 | 107,135,456 | 3,160,400 | 83,569 | 15,465,682 | 481,141 | 496,750,169 |
| *P. hypargyreus (outgroup)* | 31,742,254 | 3,197,135 | 10,286,662 | 1,527,839 | 755,592,383 | 102,813,089 | 1,414,916 | 138,744 | 13,702,193 | 508,944 | 472,143,132 |
| *P. hypargyreus (outgroup)* | 31,063,294 | 3,522,984 | 8,473,002 | 2,220,642 | 705,632,769 | 111,593,772 | 2,428,373 | 120,985 | 16,852,768 | 516,160 | 489,088,090 |
| *P. juncosae (outgroup)* | 32,273,262 | 3,105,401 | 9,409,614 | 2,444,545 | 693,803,469 | 130,848,856 | 6,276,436 | 123,900 | 16,596,184 | 583,090 | 473,539,891 |
| *P. lehmanii (outgroup)* | 32,993,418 | 4,502,257 | 10,139,156 | 1,517,571 | 728,111,475 | 101,644,208 | 3,479,471 | 167,729 | 13,929,927 | 557,946 | 500,997,539 |
| *P. nigrescens (outgroup)* | 34,376,438 | 4,452,814 | 10,168,935 | 1,961,467 | 725,187,750 | 104,723,010 | 4,808,591 | 115,476 | 13,113,239 | 550,235 | 492,328,579 |

**Supplementary Table 03** OrthoFinder analyses on 5 random *Scalesia* and 5 random outgroup genomes. Each analysis was repeated three times (runs 01-03) to assess the consistency of our results. The numbers of run 01 are also displayed in Figure 03.

| **Lineage** | **DNA** | **Helitron** | **LTR** | **SINE** | **Satellite** | **Simple repeat** |
| --- | --- | --- | --- | --- | --- | --- |
| **Run 01** |  |  |  |  |  |  |
| *Scalesia* | 43 | 15 | 791 | 11 | 1 | 2 |
| *Outgroup* | 34 | 2 | 860 | 7 | 5 | 4 |
| *Common* | 256 | 34 | 2,195 | 9 | 0 | 15 |
| **Run 02** |  |  |  |  |  |  |
| *Scalesia* | 36 | 16 | 885 | 16 | 2 | 3 |
| *Outgroup* | 42 | 3 | 781 | 13 | 6 | 5 |
| *Common* | 278 | 36 | 2,240 | 6 | 0 | 18 |
| **Run 03** |  |  |  |  |  |  |
| *Scalesia* | 32 | 14 | 890 | 14 | 1 | 4 |
| *Outgroup* | 40 | 4 | 804 | 7 | 4 | 4 |
| *Common* | 272 | 35 | 2183 | 6 | 0 | 15 |

**Supplementary Figure 01 Testing for coverage differences in running dnaPipeTE.** We started by simulating Illumina read data for three random chromosomes (12, 19, 23). We then ran DNApipeTE using 0.1×, 0.2×, 0.5×, 1×, 2× and 5× coverage (5× was only ran for chromosome 12). We report the differences in the four largest classes of TEs and repeats (Simple repeats, LTR, LINE, DNA).

**
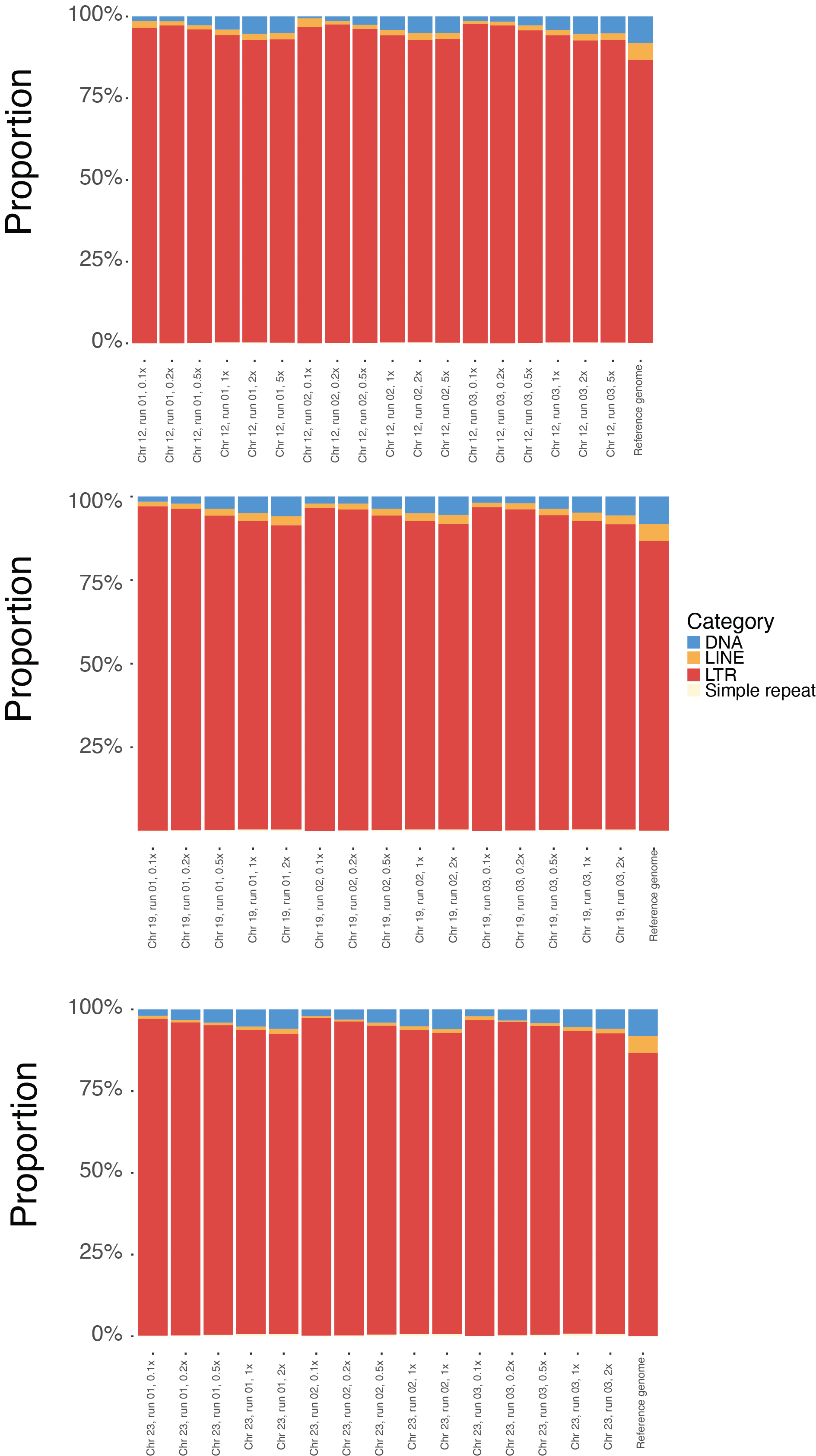
**

**Supplementary Figure 02** Stacked bar plot illustrating various TE classes (legend provided in the right part of the corner, “na*”* denotes unidentified TEs), for the analysis done with the *Scalesia-*specific TE library.
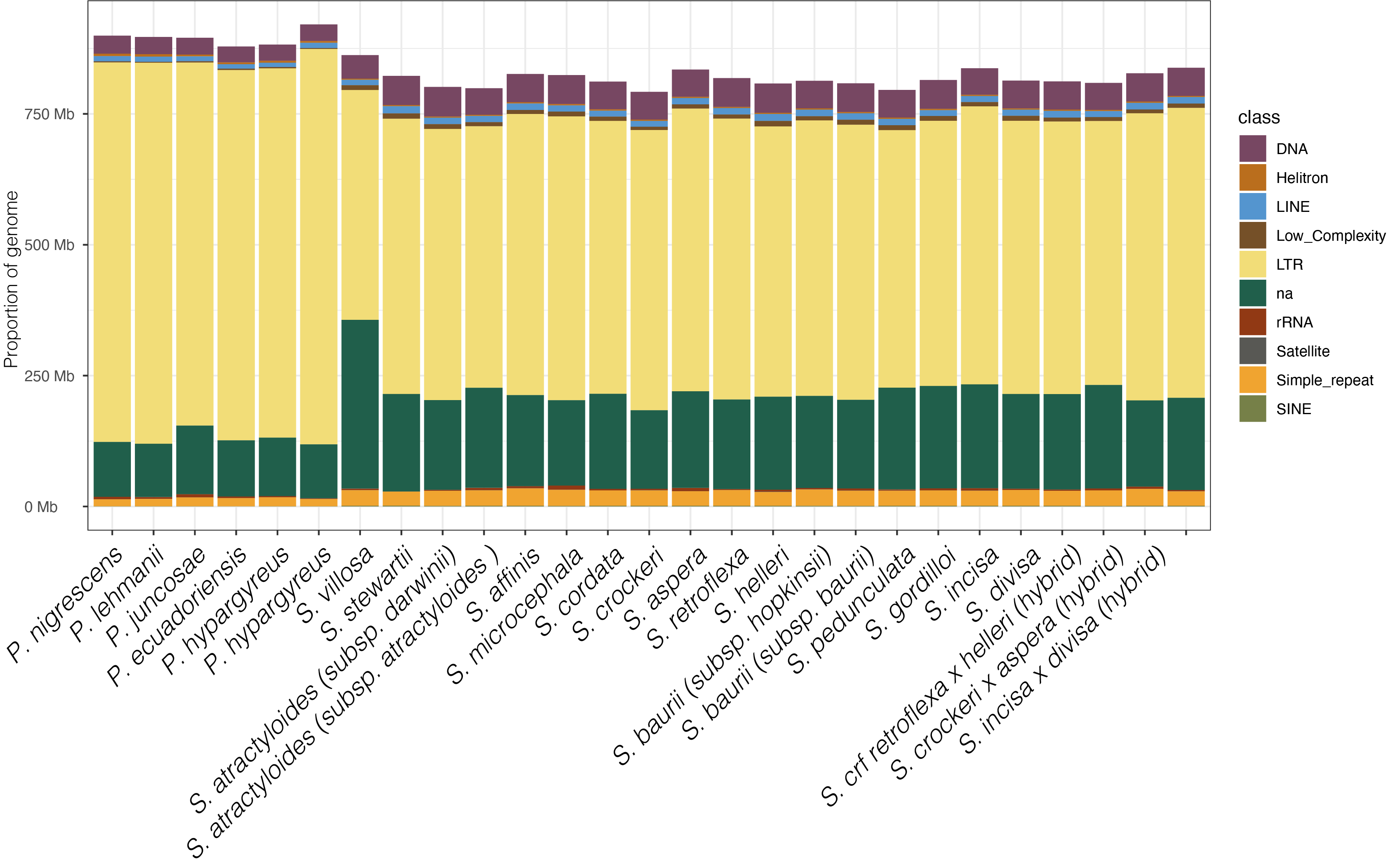


**Supplementary Figure 03 Supplementary Figure 02** Neighbor-joining phylogenetic tree of *Scalesia* radiation and the outgroup. This tree is the same as the tree presented as part of Figure 01 in the main paper, but here specimen identifiers are included.

**Supplementary Figure 04** Neighbor-joining phylogenetic tree of *Scalesia* radiation and the outgroup. This analysis includes hybrid specimens (bold).

**Supplementary Figure 05** Repeat landscape plot showing the divergence (Kimura substitution-level) of transposable elements (TEs) in the genome. This plot is similar to Figure 02, but each species is represented by a different colour.


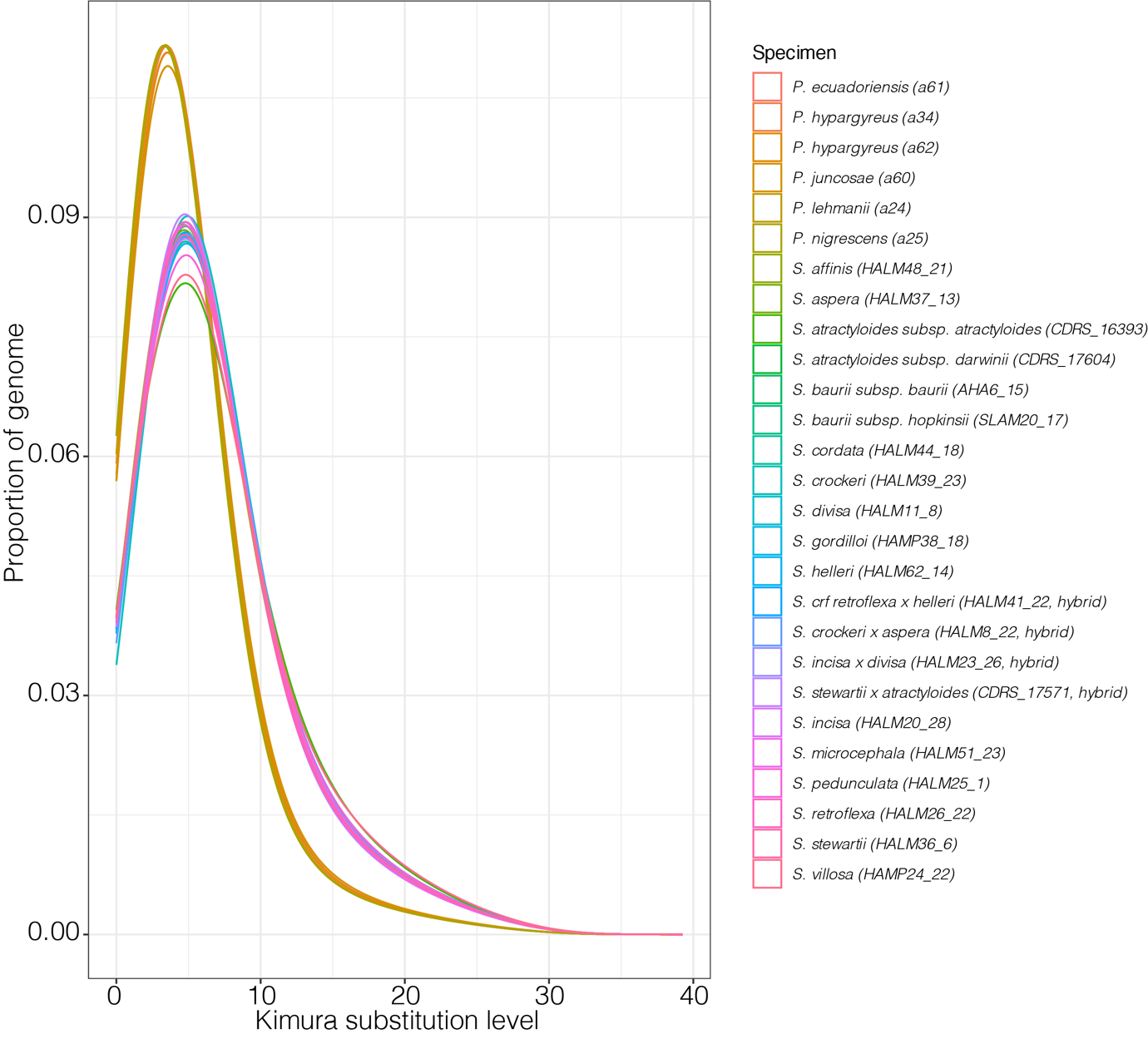


**Supplementary Figure 06** Repeat landscape plot of the *Scalesia atractyloides* reference genome.

**Supplementary Figure 07** Repeat landscape plot showing the divergence of transposable elements (TEs) in the genome for each of the outgroup specimens. Different TE groups are represented by different colors.


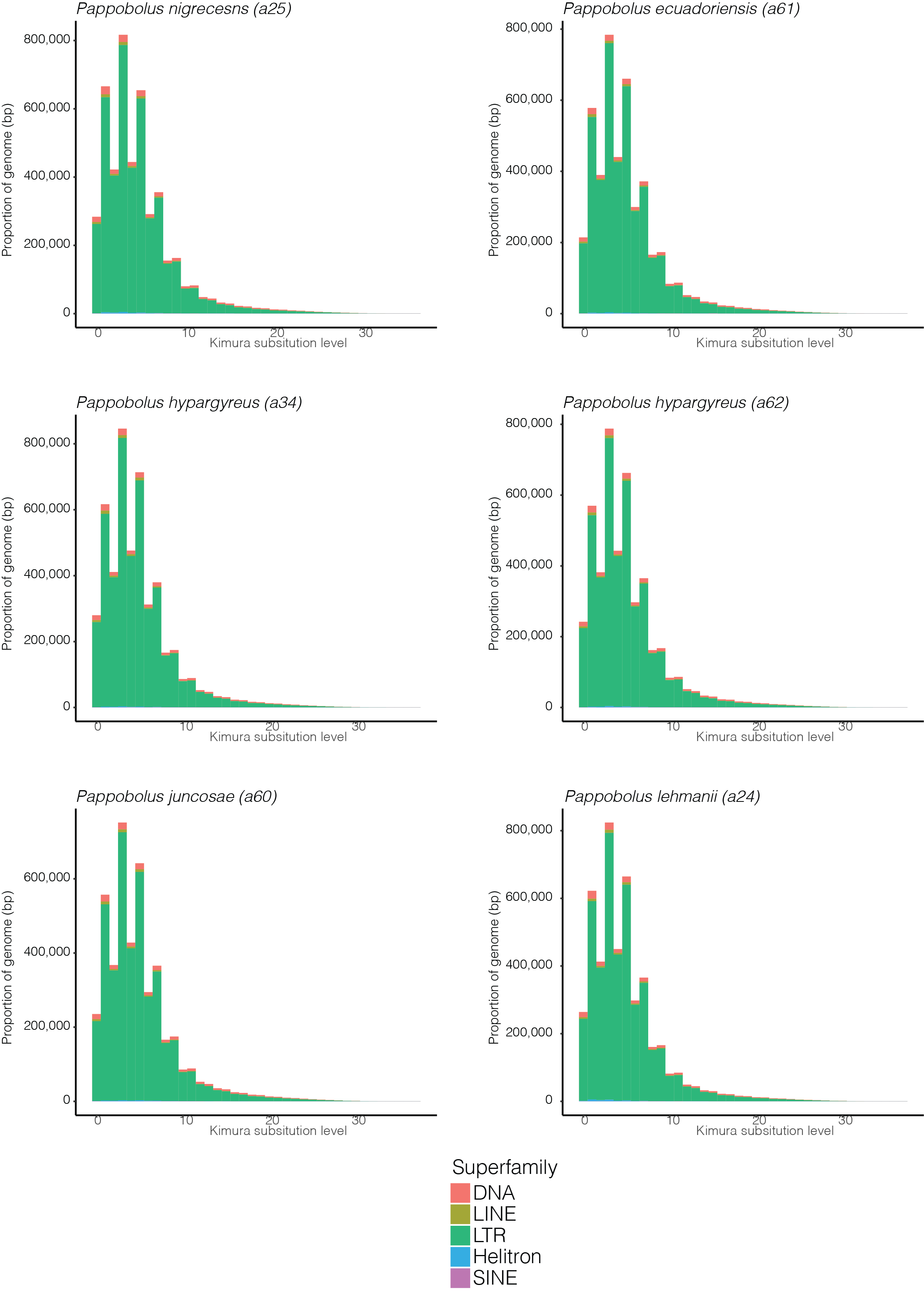


**Supplementary Figure 08** Repeat landscape plot showing the divergence of transposable elements (TEs) in the genome for *Scalesia* *affinis, S. aspera, S. atractyloides, S. baurii, S. cordata* and *S. crockeri*. Different TE groups are represented by different colors.


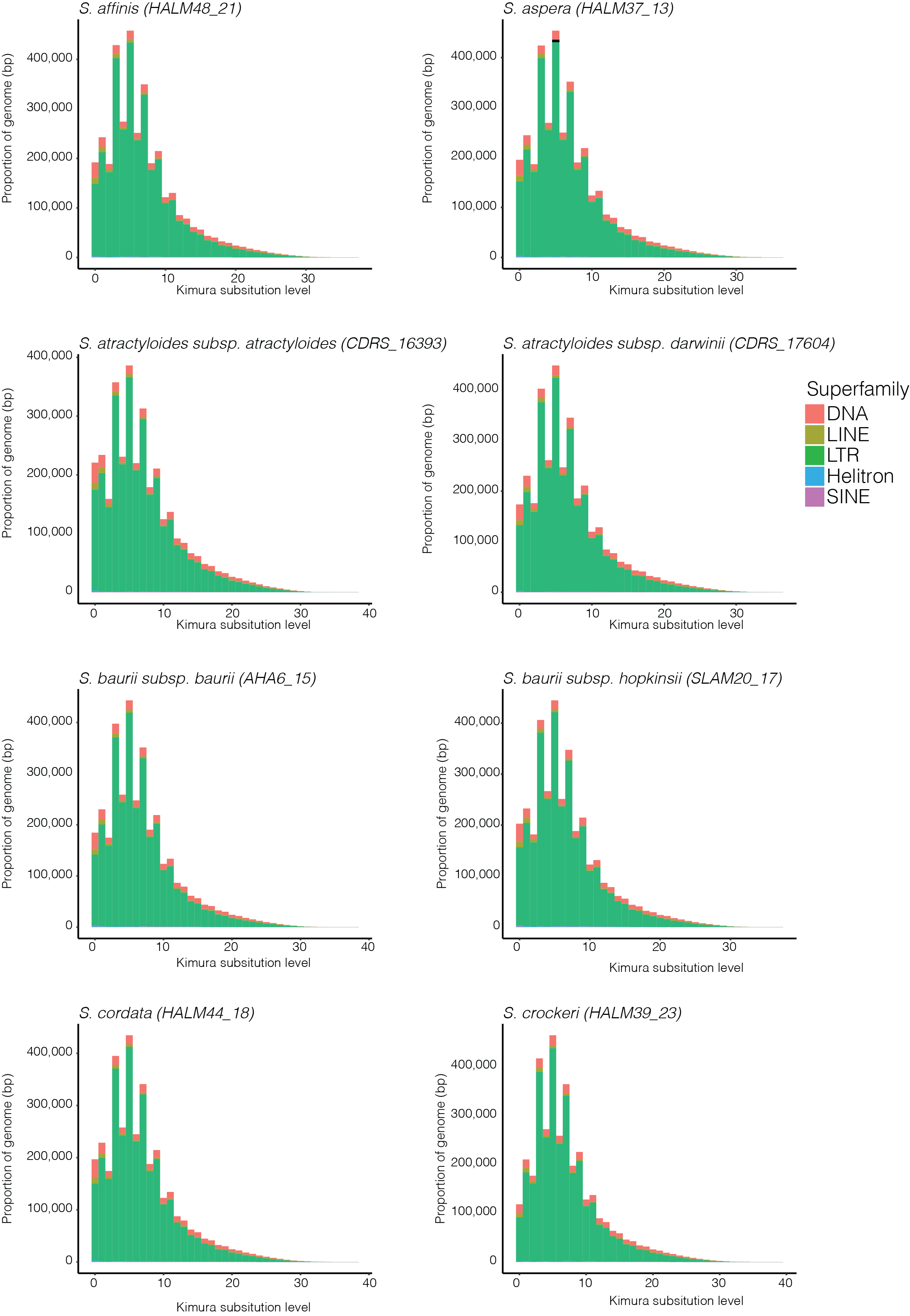


**Supplementary Figure 09** Repeat landscape plot showing the divergence of transposable elements (TEs) in the genome for *Scalesia* *divisa, S. gordilloi, S. helleri, S. incisa, S. microcephala, and S. pedunculata*. Different TE groups are represented by different colors.
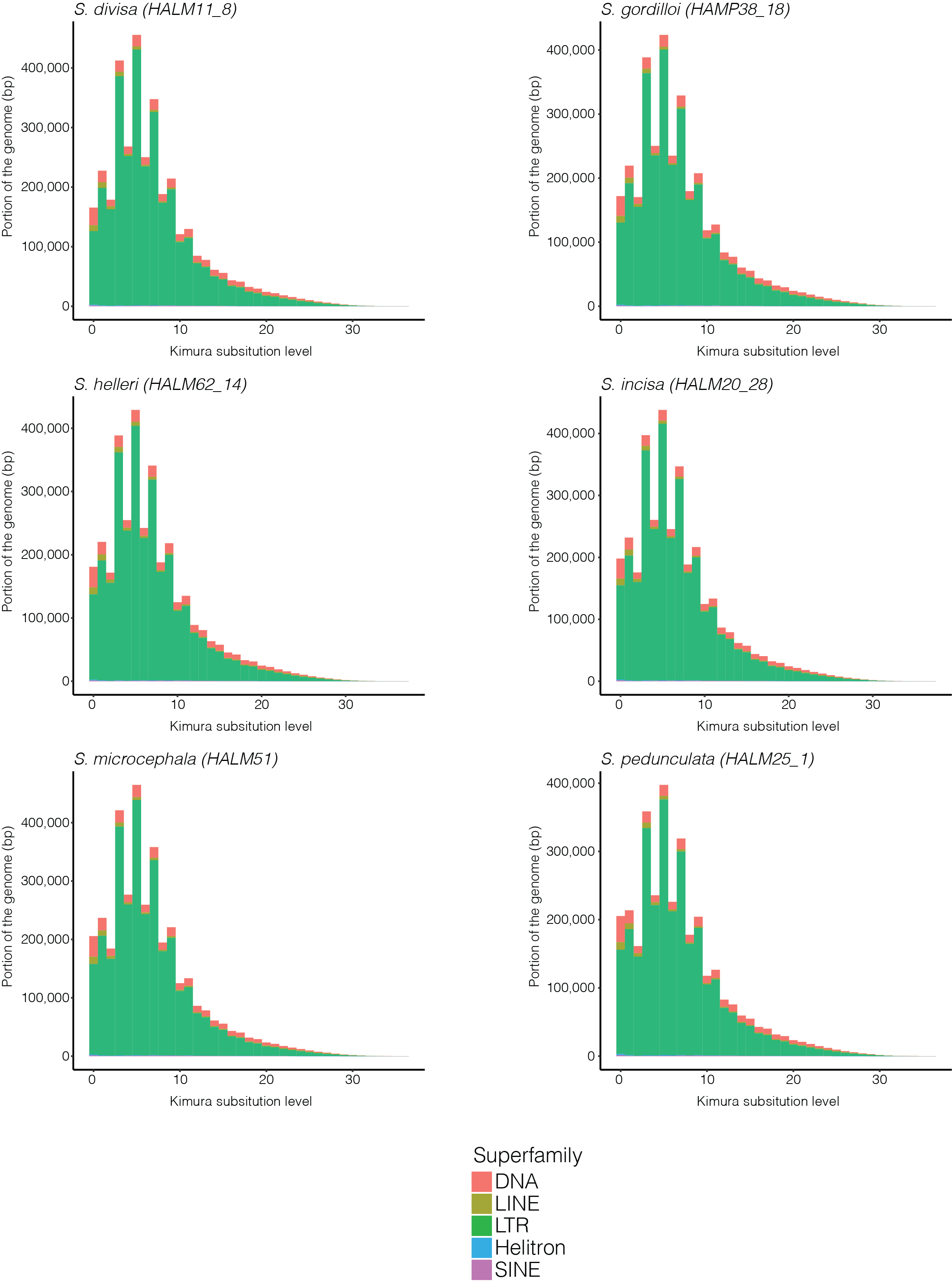


**Supplementary Figure 10** Repeat landscape plot showing the divergence of transposable elements (TEs) in the genome for *Scalesia* *retroflexa, S. stewartii, and S. villosa*. Different TE groups are represented by different colors.


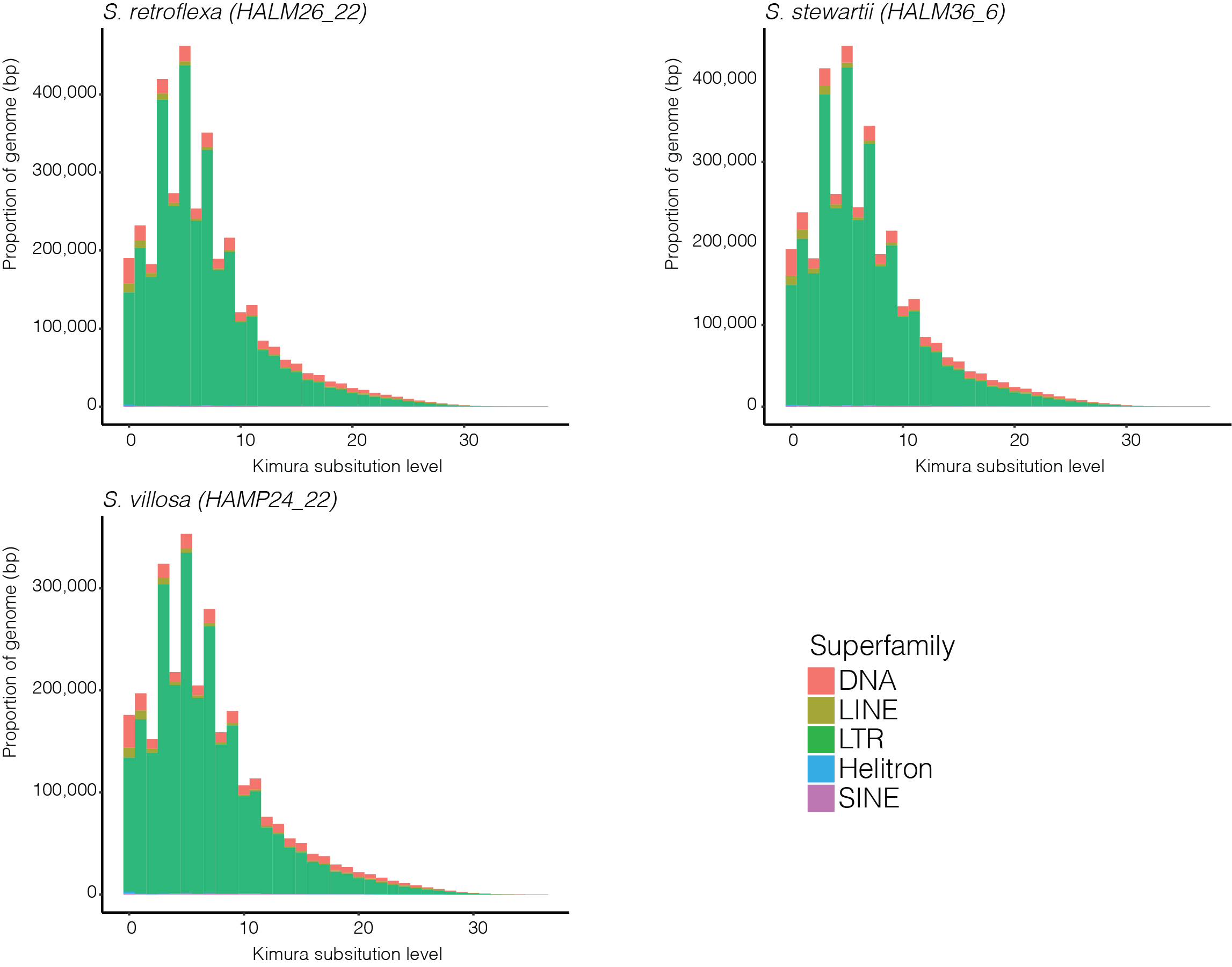


**Supplementary Figure 11** Repeat landscape plot showing the divergence of transposable elements (TEs) in the genome for *Scalesia* hybrids, including *S. incisa x divisa, S. crockeri x aspera, S. stewartii x atractyloides, S. crf retroflexa x helleri*. Different TE groups are represented by different colors.


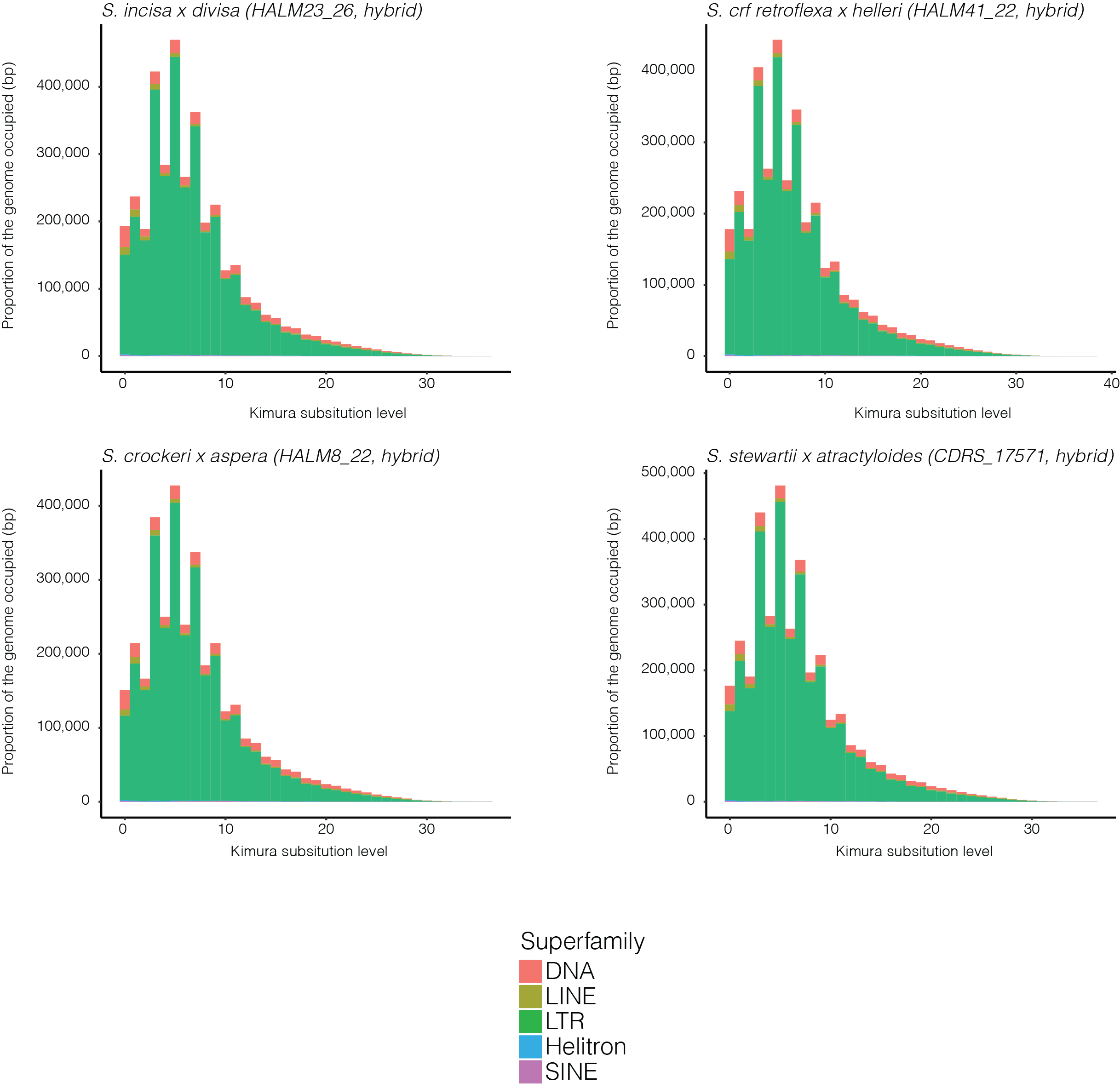


**Supplementary Figure 12** Upset plot of DNA elements, grouped in orthogroups, found within the Scalesia radiation. The number of orthogroups is plotted on the y-axis, and the x-axis shows the overlap between different genomes.


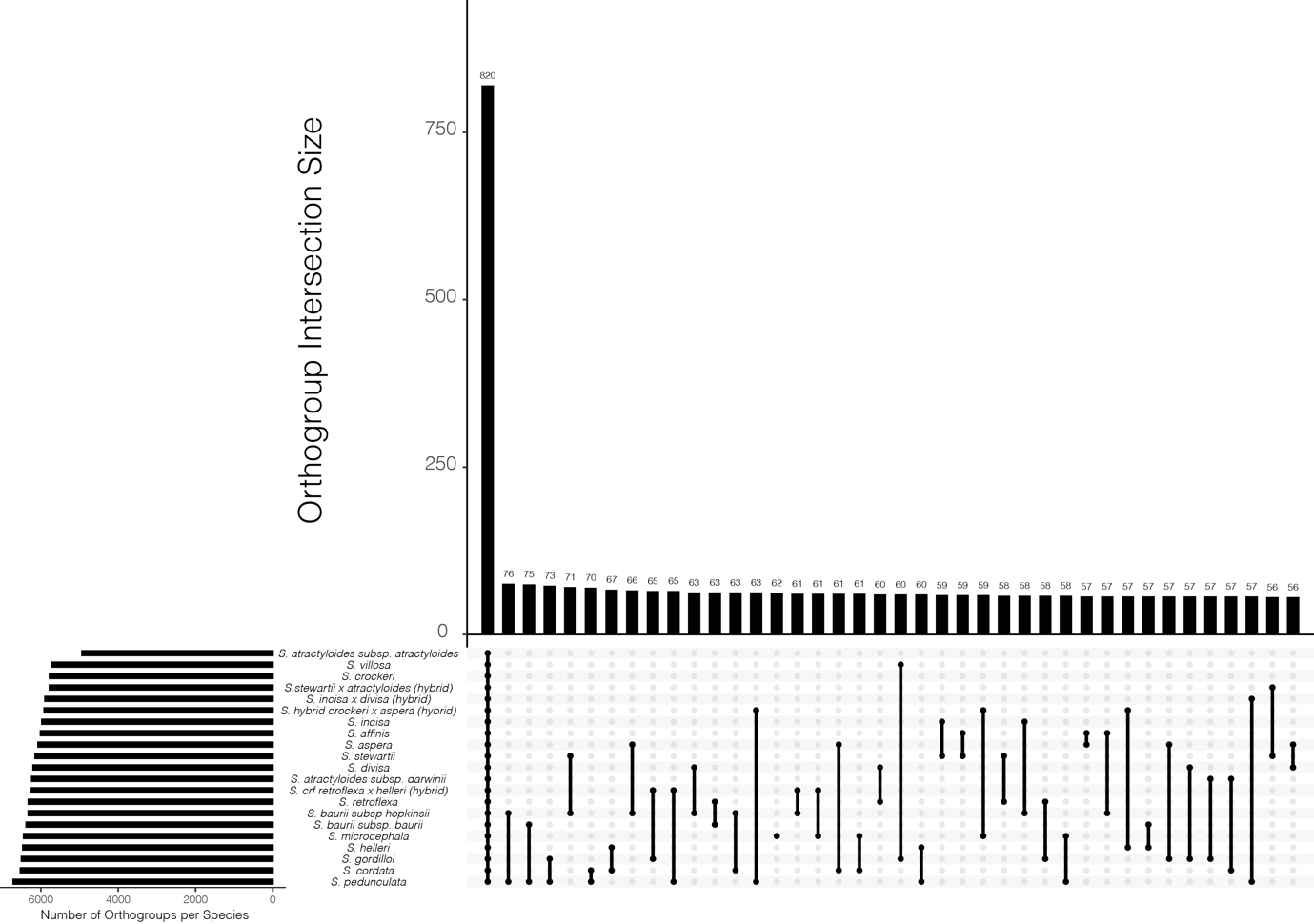


**Supplementary Figure 13** Upset plot of Helitrons, grouped in orthogroups, found within the Scalesia radiation. The number of orthogroups is plotted on the y-axis, and the x-axis shows the overlap between different genomes.


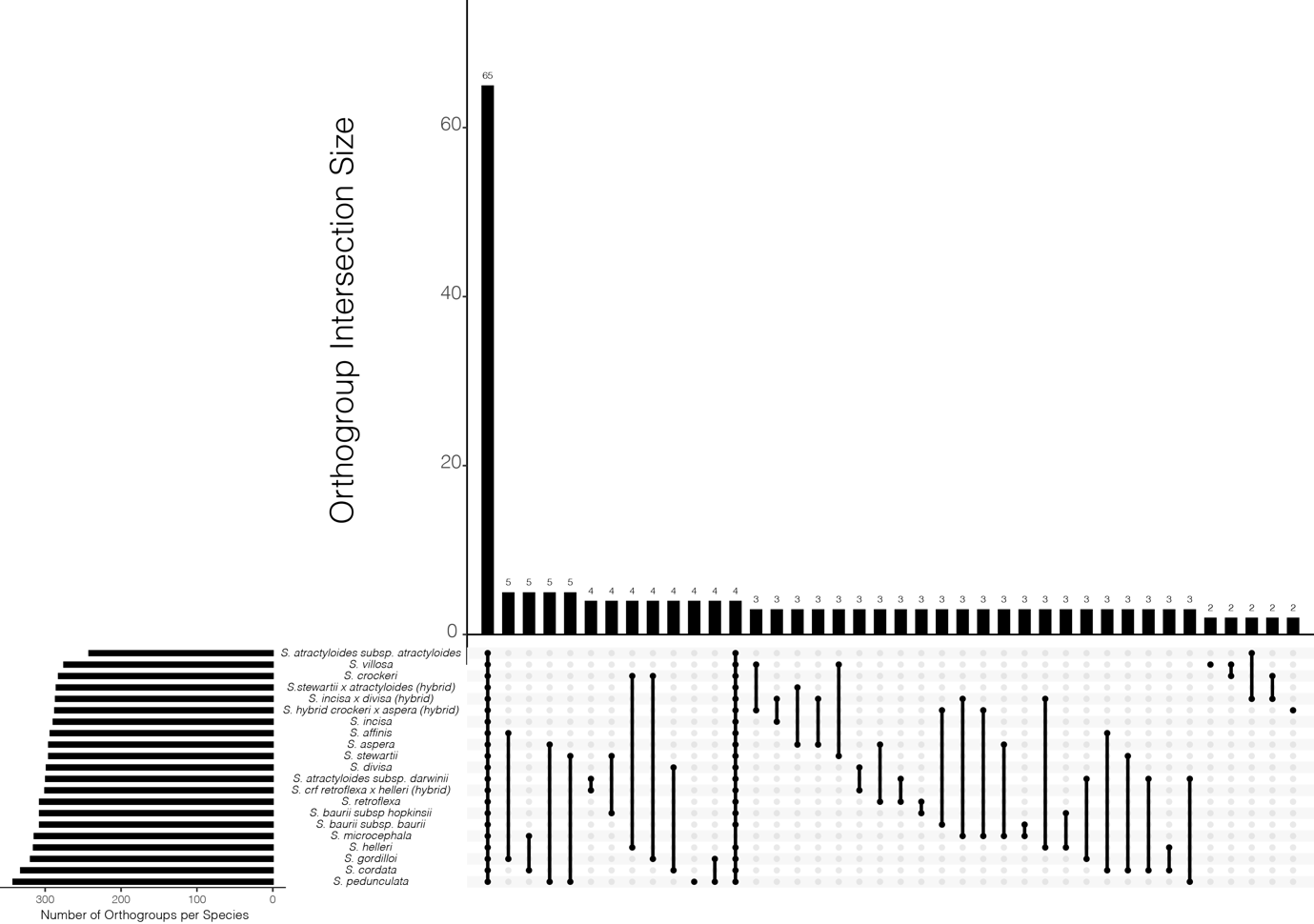


**Supplementary Figure 14** Upset plot of simple repeat elements, grouped in orthogroups, found within the Scalesia radiation. The number of orthogroups is plotted on the y-axis, and the x-axis shows the overlap between different genomes.


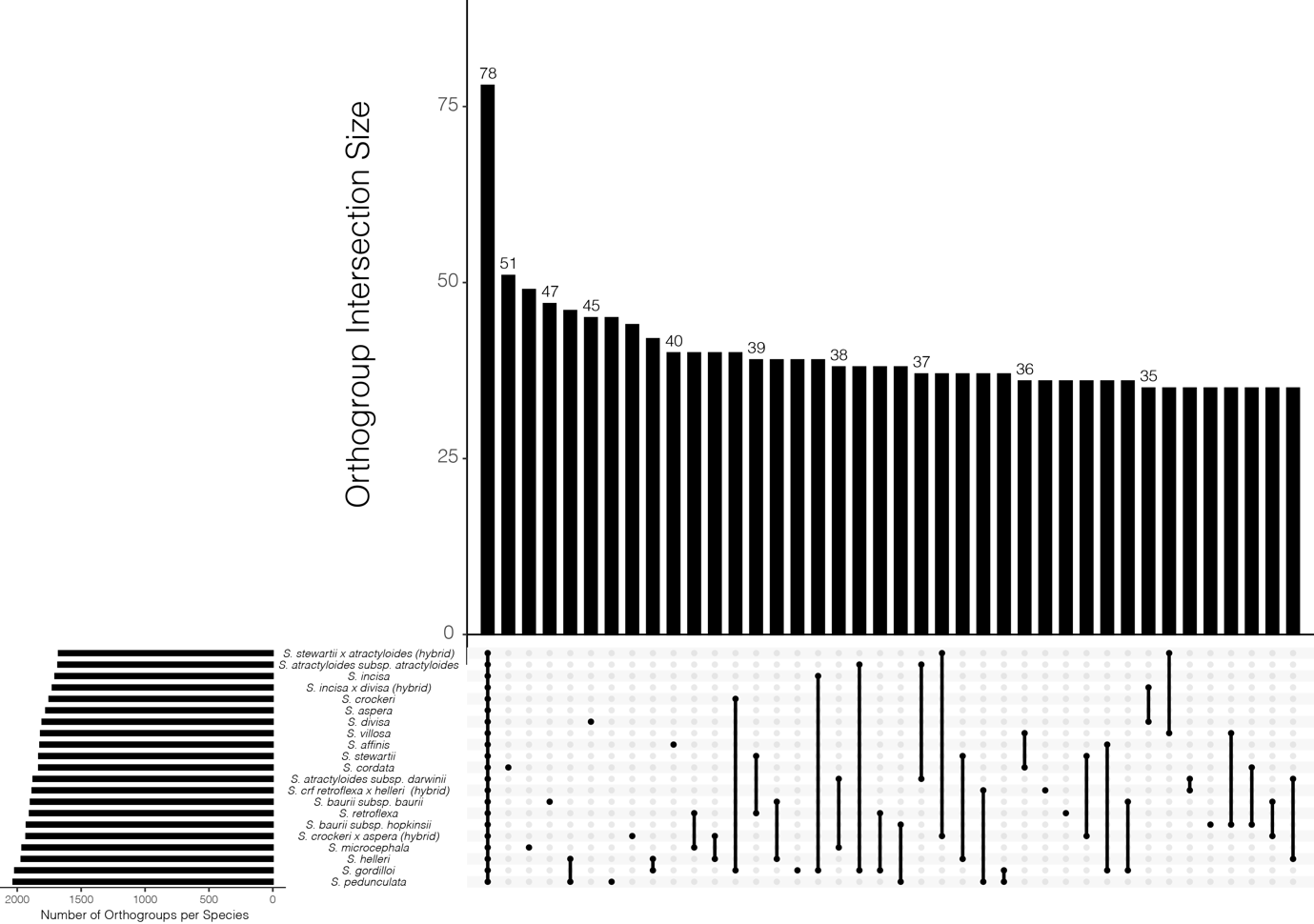


**Supplementary Figure 15** Upset plot of SINE, grouped in orthogroups, found within the Scalesia radiation. The number of orthogroups is plotted on the y-axis, and the x-axis shows the overlap between different genomes.


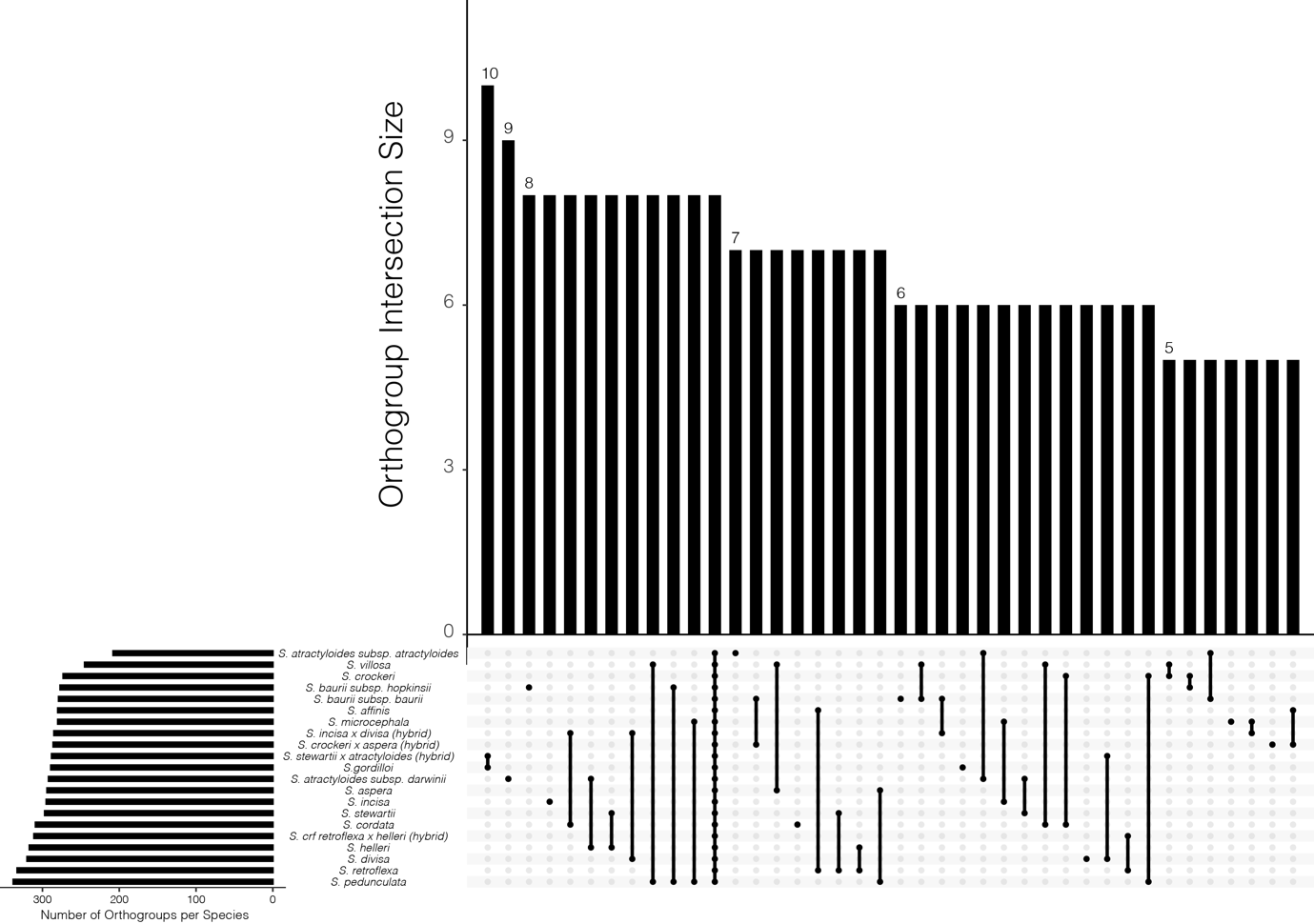


**Supplementary Figure 16** Upset plot of Satellite elements, grouped in orthogroups, found within the *Scalesia* radiation. The number of orthogroups is plotted on the y-axis, and the x-axis shows the overlap between different genomes.


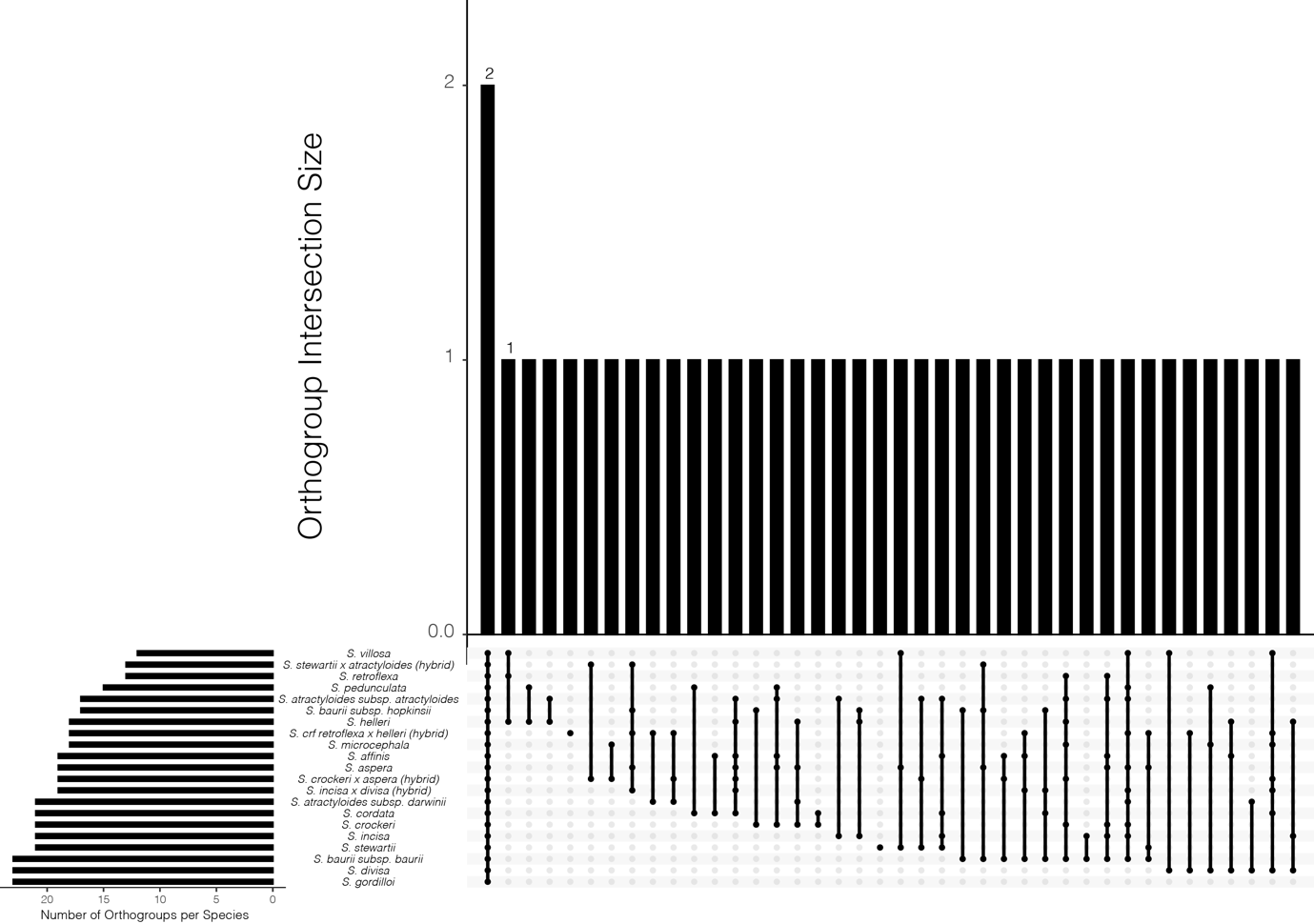

Supplement: Supplementary file 1 — Supplementary Material 1 [file 13100_2025_362_MOESM1_ESM.docx]
